# Temporary consumption of western diet trains the immune system to reduce future gut inflammation

**DOI:** 10.1016/j.isci.2023.106915

**Published:** 2023-05-20

**Authors:** Dongwen Wu, Xiaotong Wang, Xiang Yang, Lei Gu, Mandy J. McGeachy, Xiaowei Liu

**Affiliations:** 1 Department of Gastroenterology, Xiangya Hospital, Central South University, Changsha, Hunan, China; 2 National Clinical Research Center for Geriatric Disorders, Xiangya Hospital, Changsha, Hunan, China; 3 Changsha Aier Eye Hospital, Changsha, Hunan, China; 4 Department of Microbiology & Immunology, College of Veterinary Medicine, Cornell University, Ithaca, NY 14853, USA

**Keywords:** Immunology, Immune system, Immune response

## Abstract

Urbanization drives the popularity of western diet (WD), which increased burden in metabolic diseases but also in inflammatory diseases. Here, we show continuous WD disrupted the gut barrier, initiating low-grade inflammation and enhancing the colitis response. Nevertheless, transient WD consumption followed by *ad libitum* normal diet enhanced mucin production and tight junction protein expression in recovered mice. Furthermore, transient WD consumption surprisingly reduced the subsequent inflammatory response in DSS colitis and *Citrobacter rodentium*-infection induced colitis. The protective effect of WD training was not sex-dependent, and co-housing experiments suggested microbiota changes were not responsible. We identified important roles for cholesterol biosynthesis pathway and macrophages, pointing to innate myeloid training. Together, these data suggest detrimental effects of WD consumption can be reversed on return to a healthier diet. Furthermore, transient WD consumption leads to beneficial immune training, suggesting an evolutionary mechanism to benefit from feasting when abundant food is available.

## Highlights

• Short-term training by WD results in a non-specific protection from future colitis
• Mevalonate pathway is evolved in WD training independent of cholesterol biosynthesis
• Macrophages are required mediator of the protective WD training effect

## Graphical abstract

**Figure:**
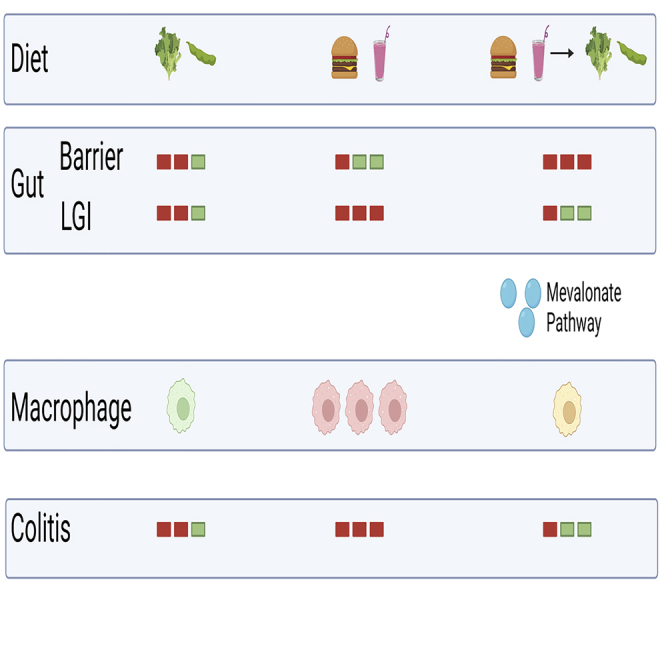

## Introduction

Diet, an important determinant of human health, used to differ among nations but global urbanization changed it in a fairly consistent ‘western diet’ (WD) way: high in refined fat and processed carbohydrates.^1^ Over past two decades, epidemiological studies have linked WD to metabolic diseases, cardiovascular diseases as well as several gastrointestinal diseases, including inflammatory bowel disease (IBD) and non-alcoholic steatohepatitis (NASH).^2^^,^^3^ At the same time, there is consensus that the recent dramatically increased incidence of IBD in developing countries is partially because of the increased prevalence of this type of diet.^4^ Moreover, accumulating preclinical evidence indicates WD promotes IBD in many ways, including gut barrier disruption,^5^ low-grade inflammation induction,^6^ and microbiome dysbiosis.^7^ Although these deleterious changes may be transient and revert on adoption of a normal diet,^8^ how and whether temporary periods of WD consumption and associated inflammatory changes affect subsequent colitis severity is unclear.

Different from T/B cell immune memory, trained immunity is referred to as immunological memory of innate immune cells, which appears as an altered reaction when encountering a secondary stimulus, either homologous or even heterologous to the first insult.^9^ Therefore, non-specific beneficial effects of immune training encouraged clinical trials that took advantage of Bacillus Calmette-Guérin (BCG) vaccination against SARS-CoV-2 infection to bridge the gap until specific COVID-19 vaccine was developed, which indicated that BCG vaccination conferred protection against COVID-19.^10^^,^^11^ Although COVID-19 vaccines were built and distributed quickly since its outbreak, the non-specific effects of vaccines and trained immunity still worth and should continue to be studied as a potential and temporary tool to reduce susceptibility and severity as well as to limit transmission in the beginning of future pandemics until specific vaccines could be adopted.^12^ Except for BCG and β-glucan (another commonly used inducer for trained immunity),^13^^,^^14^ we recently proved that immune training of gut-associated lymphoid tissues could also been achieved by low-dose dextran sodium sulfate (DSS), which modulated future adaptive responses and reduced infection-associated colitis.^15^ Given the inflammatory nation of WD, it is conceivable to find that “sterile” inflammation triggered by WD promotes granulocyte monocyte precursor cells memory establishment.^16^ However, little is known about how diet-induced training affects diseases outcomes.

It is well established that continuous WD enhances inflammatory responses to injury-induced colitis. Here, we demonstrate in mice that transient consumption of WD followed by a return to normal chow altered immune responses in the gut such that future inflammation because of injury or infection was reduced. Furthermore, this protective ‘training’ of gut-associated immunity by temporary consumption of WD was independent of gut microbe changes and occurred in both male and female mice. Mechanistically, we identified the cholesterol biosynthesis mevalonate pathway and macrophages as required mediators of the protective WD training effect. Together these results further support the benefit of adopting a healthy plant-based diet by WD consumers, because this change did not merely reverse deleterious effects of WD on gut inflammation, it revealed a beneficial immune training outcome of the prior WD-driven inflammatory response.

## Results

### WD-disrupted gut barrier is enhanced after normal diet recovery

**Figure 1:**
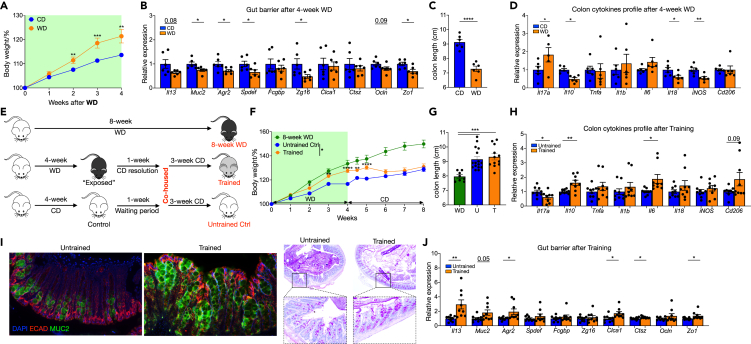
WD-disrupted gut barrier is enhanced after normal diet recovery (A–D) WT male mice were feed on WD and outcomes were analyzed 4 weeks later. (A) Body weights shown as percentage of starting weight (n = 11 to 18 per group). (B) and (D) Expression of indicated genes in distal colon tissue, normalized to *Gapdh* and shown as relative mean of control group. (C) Colon length. (E–J) WT mice were trained by 4-week ‘on and off’ WD and outcomes were analyzed after chow diet rest. (E) Experimental design. (F) Body weights shown as percentage of starting weight (n = 10 to 19 per group). (G) Colon length. (H) and (J) Expression of indicated genes in distal colon tissue, normalized to *Gapdh* and shown as relative mean of control group. (I) representative images of IF (left) and PAS staining (right) of tissue sections from distal colon. Data points represent individual mice, pooled from two independent experiments except for (C) from one experiment. All data are represented as means ± SEM. p values were calculated by Student’s *t* test or one-way ANOVA for (G). For (A) and (F), Student’s *t* test was performed independently at each time point; ∗p < 0.05, ∗∗p < 0.01, and∗∗∗p < 0.001.

Gut barrier, composed of mucus layer and epithelium tight junction proteins (TJPs) complexes, shields lamina propria and draining lymph nodes immune cells from over-activation.^5^ 16-week feeding of WD with abundant saturated fat and carbohydrates impaired gut barrier structural formation (Figures S1A and S1B) and increased bacteria dissemination to mesenteric LNs (MLNs) (Figure S1C). Barrier dysfunction has been highlighted as an initiation and promotion factor for gut inflammation.^17^^,^^18^ Indeed, mice receiving WD had shortened colons (Figure S1D), low-grade inflammation with increased gene expression for Th1 and Th17 markers (Figure S1E) and skewed cytokines profiles (Figure S1F). Nevertheless, the gene expression of *Il22* and it induced antimicrobial peptide, e.g., RegIIIβ and RegIIIγ, shown no difference between group (Figure S1G). Concomitant with local low-grade inflammation, markers of immune activation were also observed in MLNs (Figure S1H). Of note, 4-week WD phenocopied the weight gain (Figure 1A), lower gut barrier molecules expression (Figure 1B) and higher *Il17a* but lower *Il10* expression (Figures 1C and 1D) observed in long-term WD, demonstrating short-term WD feeding is sufficient to drive gut changes.

The intestinal barrier is a rapidly self-renewing structure with a turnover period of 4–5 days for epithelial cells (5–7 days in human) and fast renewal of the mucus layers by the surface goblet cells.^19^ WD is known to disrupt the gut barrier and evoke low-grade inflammation, but whether the barrier is repaired and whether low-grade inflammation is resolved after normal chow diet rest has yet to be explored. To tackle this question, we employed a training model by temporary WD exposure followed by 1-week normal chow resolution. To further rule out contributions of microbiota changes to any observed effects we then co-housed the WD-fed mice with control mice that had received with normal chow for a further 3 weeks (Figure 1E). Mice switched to normal diet gained less weight than those maintained on WD throughout (Figure 1F). Switching to normal diet also reversed colon shortening (Figure 1G). After normal diet “wash-out”, WD exposed mice (hereafter termed trained mice) had comparable body weight and colon length to control untrained mice (Figures 1F and 1G). Intriguingly, colon cytokines profile revealed markedly higher *Il10* but lower *Il17a expression* in trained mice (Figure 1H), indicating an anti-inflammatory situation. In addition, trained mice revealed an enhanced intestinal barrier function: there were more goblet cells, thicker mucus (Figure 1I) and increased expression of *Il13*, core mucus structural components (e.g., *Muc2, Clca1*, and *Ctsz*) and TJPs (Figure 1J) in distal colon compared with untrained mice.

### WD training provides protection on injury-induced colitis

**Figure 2:**
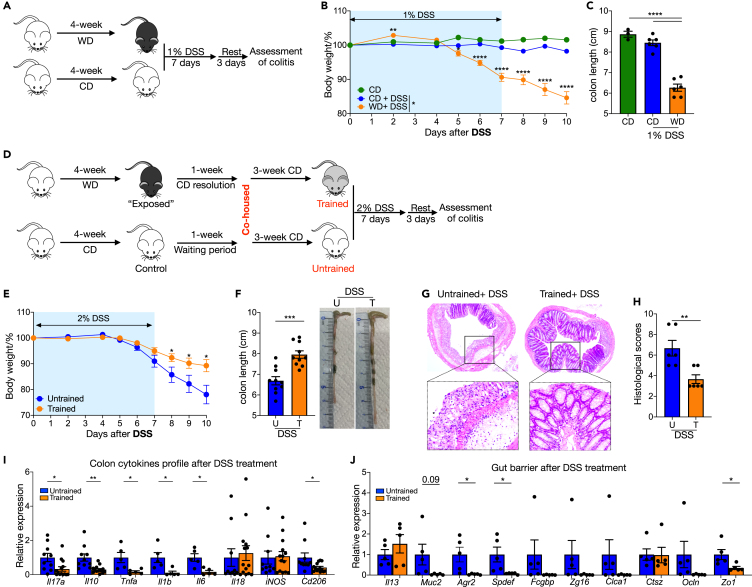
WD training provides protection on injury-induced colitis (A–C) WD-fed mice and chow diet male mice received 1% DSS in drinking water for 7 days to induce mild inflammation, a control group received normal drinking water. After 3 days of rest (normal drinking water), mice were sacrificed. (A) Experimental design. (B) Body weights shown as percentage of starting weight (n = 9 to 15 per group). (C) Colon length. (D–J) WT male mice were trained by 4-week ‘on and off’ WD, a control group received normal chow diet and drinking water. Then 2% DSS in drinking water were administered for 7 days to induce inflammation followed by 3 days recovery. (D) Experimental design. (E) Body weights shown as percentage of starting weight (n = 11 to 15 per group). (F) Colon length and method for quantifying colon length. (G) representative images of H&E of tissue sections from distal colon. (H) Histological scores from multiple mice. (I) and (J) Expression of indicated genes in distal colon tissue, normalized to *Gapdh* and shown as relative mean of control group. Data points represent individual mice, pooled from two independent experiments. All data are represented as means ± SEM. p values were calculated by Student’s *t* test or one-way ANOVA for (C). For (B) and (E), Student’s *t* test was performed independently at each time point; ∗p < 0.05, ∗∗p < 0.01, and∗∗∗p < 0.001.

The data so far suggest that WD feeding damages the gut barrier, and that subsequent inflammatory response and repair results in potentially enhanced barrier function. To test these concepts, we challenged mice receiving WD or that had been previously trained with transient WD with DSS to induce colitis. In a low-dose (1%) DSS treatment model (Figure 2A), mice maintained on WD showed increased weight loss (Figure 2B) and significantly shorter colon (Figure 2C) compared to mice receiving normal diet. Those results are in accordance with previous *in vivo* findings^7^ as well as observational studies from human population that WD or obesity is an important risk factor for IBD.^3^

We next tested the response of trained mice to a dose of DSS that induces frank inflammation and weight loss in normal diet fed mice (2% DSS) (Figure 2D). In agreement with markers for better gut barrier function and reduced inflammation, mice that received transient WD training showed less body mass loss (Figure 2E) and longer colons (Figure 2F). Histological analysis of distal colon confirmed reduced epithelium damage (Figure 2G) and lower histologic score (Figure 2H) after DSS treatment in trained mice. Furthermore, colon from those trained mice exhibited lower *Il17a, Il10, Tnfa, Il1b, Il6 and Cd206* expression (Figure 2I). Barrier related genes expression, especially goblet cell-specific genes (e.g., *Muc2*, *Agr2* and *Spdef*), were relatively high at mRNA level in DSS treated untrained mice (Figure 2J), which may suggest a compensatory mechanism for destruction. These data support the conclusion that consumption of WD causes inflammation and barrier dysfunction that can be repaired on return to a healthier normal diet, and in fact this transient diet-driven inflammatory response provides future benefits to gut health by increasing resistance to injury-driven colitis.

### WD training nonspecifically protects mice from colitis

**Figure 3:**
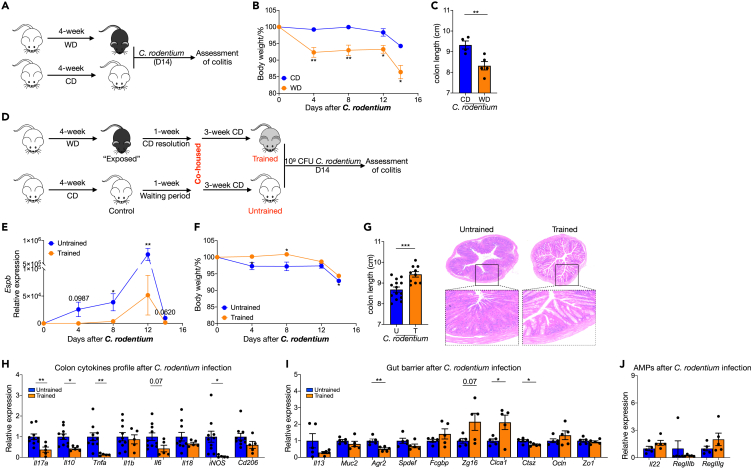
WD training nonspecifically protects mice from colitis (A–C) WD-fed mice and chow diet male mice were infected with *C. rodentium* by oral gavage, and outcomes were analyzed on day 14 after infection. (A) Experimental design. (B) Body weights shown as percentage of starting weight (n = 4 to 5 per group). (C) Colon length. (D–I) WT male mice were trained by 4-week ‘on and off’ WD, a control group received normal chow diet and drinking water. All mice were infected with *C. rodentium* and outcomes were analyzed on day 14 after infection. (D) Experimental design. (E) *C. rodentium* count in feces (n = 10 per group). (F) Body weights shown as percentage of starting weight (n = 10 to 14 per group). (G) Colon length and representative images of H&E of tissue sections from distal colon. (H–J) Expression of indicated genes in distal colon tissue, normalized to *Gapdh* and shown as relative mean of control group.Data points represent individual mice. (B) and (C) from one experiment and (E–J) were pooled from two independent experiments except. All data are represented as means ± SEM. p values were calculated by Student’s *t* test. For (B), (E), and (F), Student’s *t* test was performed independently at each time point; ∗p < 0.05, ∗∗p < 0.01, and∗∗∗p < 0.001.

Exacerbation of colitis severity by WD consumption has been reported in other colitis models.^20^ *Citrobacter rodentium* (*C. rodentium*) is an attaching and effacing (A/E) bacterial pathogen that mimics human diarrheagenic enteropathogenic *Escherichia coli* (EPEC) and enterohemorrhagic *E*. *coli* (EHEC).^21^ Importantly, *C. rodentium* causes a transient colitis before it is cleared by 2–3 weeks post-infection.^22^ We confirmed that WD-fed mice had more severe colitis outcomes during *C. rodentium* infection (Figures 3A–3C).

Considering the benefits of prior WD training on DSS injury-induced colitis, we queried if the same protection could be generated in infection-related colitis (Figure 3D). Bacterial burden monitoring showed trained mice had significantly lower *C. rodentium* load beginning at early stages of infection (Figure 3E). Lower pathogen burden was associated with significantly less reduction in body mass (Figure 3F), longer colon and less damage (Figure 3G) after infection, indicating reduced colitis in trained mice. Gene expression analysis of trained mice colon further supported a reduced local inflammatory response compared with untrained mice, with lower expression of *Il17a, Il10, Tnfa, Il6*, and *iNOS* (Figure 3H). Although *Clca1* expression was higher, goblet cell-specific genes and *Ctsz* expression was lower in trained mice after infection (Figure 3I). However, the gene expression of *Il22*, Reg3β, and Reg3γ was not influenced by WD training (Figure 3J)

The aforementioned findings demonstrate that WD also induced mild colitis. Therefore, we tested if WD training also works for future WD challenge (Figure S2A). Trained mice had a trend to lose weight during secondary round of WD but did not reach significance (Figures S2B and S2C). Besides, colon length (Figure S2D), colon inflammatory markers (Figure S2E) and colon barrier markers (Figure S2F) were all comparable between trained and untrained mice.

Overall, despite these different pathogenic mechanisms, we found that better outcomes after WD training are common in injury-induced and infection-related colitis model.

### WD training protection is independent of gut microbe changes

Gut microbiota are sensitive to diet, and WD high in fat but low in fiber lacks nutrients to support gut microbiota diversity.^20^ In addition, microbial dysbiosis is proposed to be a central mechanism for how WD exacerbates colitis.^23^ We therefore queried if gut flora changes contributed to WD training induced protection on colitis. Co-housing of mice is known to allow sharing of microbiota populations between the mice. Previously WD-trained mice were protected from subsequent colitis whether they were co-housed or not co-housed with control mice that had only received normal chow diet (Figures S3A–S3D).

**Figure 4:**
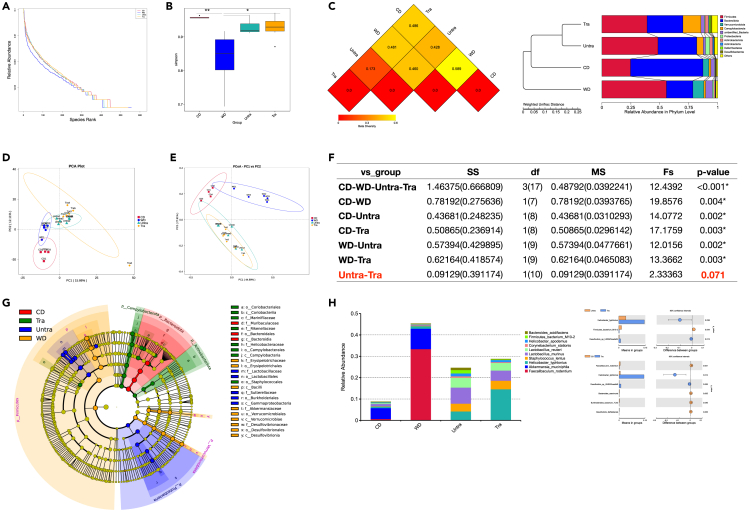
WD training protection is independent of gut microbe changes (A–H) Fecal samples were collected from chow diet (4 mice), 8-week WD (5 mice), untrained (6 mice) and trained mice (6 mice) and did 16s rRNA sequencing. (A) Rank Abundance Curve for Alpha diversity. (B) *Simpson* Diversity Index for Alpha diversity. (C) Weighted unifrac distance for Beta diversity. (D) Principal Component Analysis. (E) Principal Co-ordinates Analysis. (F) Analysis of molecular variance (Amova) of Unifrac Distance. (G) Linear discriminant analysis (LDA) effect size (LEfSe). (H) Relative abundance at species level. p values were calculated by Turkey test; ∗p < 0.05, ∗∗p < 0.01, and∗∗∗p < 0.001.

To define the composition of gut microbiota, we performed fecal 16s rRNA sequencing among chow diet, 8-week WD, untrained and trained mice (Figure 1E). Consist with previous publications,^24^ WD resulted in a substantial reduction in microbiota Alpha diversity by Rank Abundance Curve (Figure 4A) and *Simpson* and *Shannon* Diversity Index (Figures 4B and S3E), but no significant difference was found among chow diet, untrained and trained mice on Alpha diversity. Based on weighted unifrac distance, minimal difference was found between trained and untrained mice whereas WD mice showed general disruption of the fecal bacterial community structure to chow diet mice (Figures 4C and S3F). Later Principal Component Analysis (Figure 4D) and Principal Co-ordinates Analysis (Figure 4E) further confirmed comparable Beta diversity between trained and untrained mice. Analysis of molecular variance (Amova) of Unifrac Distance showed the difference between trained and untrained mice did not reach significance (Figure 4F). Best discriminated taxa in each groups was presented as Linear discriminant analysis (LDA) effect size (LEfSe) (Figure 4G), and difference was tested by species level (Figure 4H). Although there were a few differences (e.g., *Helicobacter typhlonius*), overall trained mice and untrained mice harbor similar gut microbes. Altogether, no obvious difference in gut flora between trained and untrained mice suggested the training benefits are not driven primarily by changes in gut microbes.

### WD training improves colitis outcome through mevalonate pathway but is independent of serum cholesterol level

**Figure 5:**
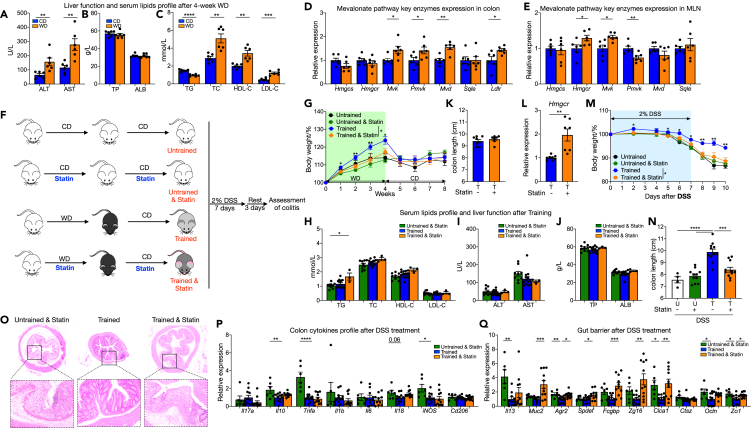
WD training improves colitis outcome through mevalonate pathway but is independent of serum cholesterol level (A–E) WT male mice were feed on WD and outcomes were analyzed 4 weeks later. (A) Serum alanine aminotransferase (ALT) and aspartate aminotransferase (AST). (B) Serum total protein (TP) and albumin (ALB). (C) Serum total glyceride (TG), total cholesterol (TC), high-density lipoprotein cholesterol (HDL-C) and low-density lipoprotein cholesterol (LDL-C) level. (D) and (E) Expression of indicated genes in distal colon tissue (left) and MLN (right), normalized to *Gapdh* and shown as relative mean of control group. (F–K) Trained and untrained male mice received Fluvastatin whole training and rest period and outcomes were analyzed after chow diet rest. (F) Experimental design. (G) Body weights shown as percentage of starting weight (n = 9 to 19 per group). (H) Serum TG, TC, HDL-C and LDL-C level. (I) Serum ALT, AST. (J) Serum TP, ALB. (K) Colon length. (L) Expression of *Hmgcr* in distal colon tissue, normalized to *Gapdh* and shown as relative mean of control group. (M–Q) Trained and untrained male mice received Fluvastatin whole training and rest period. Then 2% DSS in drinking water were administered for 7 days to induce inflammation followed by 3 days recovery. (M) Body weights shown as percentage of starting weight (n = 4 to 11 per group). (N) Colon length. (O) representative images of H&E of tissue sections from distal colon. (P) and (Q) Expression of indicated genes in distal colon tissue, normalized to *Gapdh* and shown as relative mean of control group. Data points represent individual mice, pooled from two independent experiments. All data are represented as means ± SEM. p values were calculated by Student’s *t* test or one-way ANOVA for (N–L). For (G) and (M), Student’s *t* test was performed independently at each time point; ∗p < 0.05, ∗∗p < 0.01, and ∗∗∗p < 0.001.

Except for gut microbiota dysbiosis, WD also leads to metabolic changes. Being the biggest metabolic organ, the liver plays a vital role for lipid metabolism and protein synthesis (especially albumin synthesis). It is well known that WD is associated with non-alcoholic fatty liver disease (NAFLD) which can further progress to NASH.^2^ Even after only 4 weeks WD feeding, serum levels of alanine aminotransferase (ALT) and aspartate aminotransferase (AST) were elevated significantly (Figure 5A), indicating the injury of hepatocytes. At the same time, though total protein and albumin levels shown no difference, increased cholesterol level but deceased triglyceride level were found after short-term WD feeding (Figures 5B and 5C), which were further confirmed in 8-week WD fed mice (Figure S4A). In addition, the expression of key enzymes in cholesterol biosynthesis mevalonate pathway (Figure S4B), like HMG-CoA reductase (coded by *Hmgcr*), mevalonate kinase, phosphomevalonate kinase, as well as mevalonate biphospho decarboxylase, changed markedly after WD feeding in both colon and MLNs (Figures 5D and 5E), indicating an adaptation for increased fat and carbohydrates intake. Based on those results, we used statin, an inhibitor for mevalonate pathway rate-limiting enzyme HMG-CoA reductase, in drinking water to prevent WD accompanied hypercholesterolemia in our training model (Figure S5A). For mice receiving chow diet, statin treatment made no influence on weight gaining, but statin reduced weight gaining for WD feeding mice (Figures 5F, 5G, S4C, and S4D). However, statin treatment had no beneficial on WD induced colon inflammation as measured by colon length (Figure S4E). Later, when DSS was introduced (Figure S5A), neither body weight (Figure S5B) nor colon length (Figure S5C) showed any difference among trained mice with or without statin treatment during training period. Cytokines in colon also had similar expression (Figure S5D), but barrier marker expression was reversed (Figure S5E). Similarly, 4-week statin treatment did not reverse the protective effects in the *C. rodentium* colitis model (Figures S6A–S6E). These results suggest that WD induced colon low-grade inflammation and later training benefits are independent of enhanced cholesterol metabolism during WD feeding.

Altered cholesterol metabolism has been associated with innate immune cell training to inflammatory stimuli, and mevalonate is important for trained myeloid cells to more effectively combat a new infection.^25^ To fully elucidate if the mevalonate pathway involved in WD training, we treated mice with statin throughout the training and rest period (Figure 5F). Despite statin treatment, body weight (Figure 5G) and serum AST, ALT, TP, ALB, and lipids (Figure 5H) were comparable among trained and untrained mice after the chow diet rest. Unexpectedly, liver function and serum lipids profile analyses demonstrated that WD only led to a temporary liver cell injury and hypercholesterolemia that quickly recovered during the 4-week rest period on normal chow, even without statin treatment (Figures 5H–5J). Furthermore, extending statin treatment made no difference to baseline colon length (Figure 5K), as well as inflammatory and intestinal barrier markers (Figures S7A–S7C), except for a relatively higher *Hmgcr* expression (Figure 5L). However, when challenged with DSS, training benefits were totally abrogated in statin-treated WD trained mice: obvious weight loss (Figure 5M) and severe colitis revealed that those mice had similar outcomes with untrained mice (Figures 5N–5Q). Together, these data indicate that the mevalonate pathway is essential for WD-induced training protection on subsequent colitis.

### Macrophages are critical for colitis protection by transient WD

**Figure 6:**
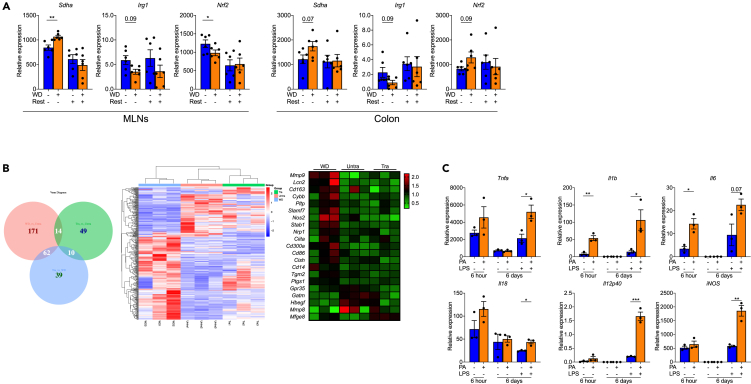
Saturated fat induced trained immunity in macrophages (A) WT mice were fed on 4-week WD or were trained by 4-week ‘on and off’ WD. Expression of indicated genes in MLNs and distal colon tissue were normalized to *Gapdh* and shown as relative mean of control group. (B) Colon tissues harvested from CD, WD and trained mice were subjected to RNA sequencing analysis. The levels of macrophages-associated genes were analyzed. (C) Macrophages RAW264.7 were treated with PA for 6 h. After 6-day rest, macrophages were re-stimulated with LPS for 24h. Expression of indicated genes were normalized to *Gapdh* and shown as relative mean of control group. Data points represent individual mice, pooled from two independent experiments except for (A). All data are represented as means ± SEM. p values were calculated by Student’s *t* test; ∗p < 0.05, ∗∗p < 0.01, and ∗∗∗p < 0.001.

The mevalonate pathway plays an essential role for the induction of trained immunity by inflammatory stimuli. Of interest, it is not thought to be the synthesis of cholesterol but rather its intermediate product mevalonic acid that is critical to induce trained immunity. Considering the non-specific protection (a main characteristic of trained immunity) of WD training, and that it appears to be dependent on the mevalonate pathway but not the levels of systemic cholesterol, we hypothesized that trained immunity may act as a key mechanism in WD training induced protection on colitis. Recently, Christ et al. shown that WD truly established long-lasting memory in *Ldlr*^*−/−*^ mice myeloid cells, which increased proliferation and enhanced innate immune responses *in vitro* even mice were shifted back to chow diet.^16^ As the important role of IRG1-itaconate-SDH axis in the development of immune training was demonstrated by Domínguez-Andrés et al.,^26^ we tested genes expression, including *Sdha, Irg1*, and Nfe2l2 (coding NRF2), in our WD training model where a similar training phenotype was confirmed (Figure 6A).

Next, to understand the mechanisms of WD training, RNA-seq was performed. We harvested colon tissues from CD, WD, and trained mice. Notably, we found a significant changes in the colons of mice fed on WD compared with CD mice, and those changes was markedly restored in training mice, especially for macrophages marker genes (Figure 6B). RT-PCR further confirmed our RNA sequencing findings that shown no matter of long-term 16-week (Figures S8A and S8B) or short-term 4-week WD (Figures S8C and S8D) changed macrophage polarization significantly in both colon and MLNs. However, those macrophages markers were comparable between trained and untrained mice (Figure S8E), suggesting the macrophage activation status returns to the baseline state from primed immune responses (a critical point for trained immunity^27^). Next, the training effects of saturated fat on macrophages was explored by using palmitic acid (PA) *in vitro*. Although cytokines production came back to normal level after rest, PA pre-treated macrophages shown stronger phenotype during LPS re-challenge (Figure 6C), suggesting macrophages might be involved in our WD training model.

**Figure 7:**
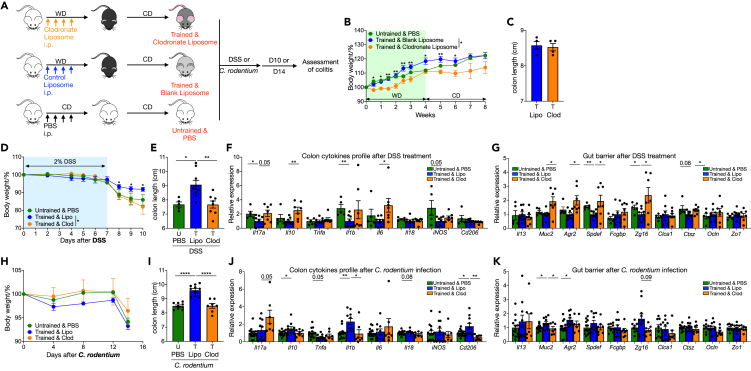
Macrophages are critical for colitis protection by transient WD (A–C) Trained male mice received liposome-encapsulated clodronate or blank control liposome i.p. injection, an untrained control group received PBS i.p. injection. (A) Experimental design. (B) Body weights shown as percentage of starting weight (n = 7 to 9 per group). (C) Colon length. (D–G) Trained and untrained male mice received liposome or PBS injection. Then 2% DSS in drinking water were administered for 7 days to induce inflammation followed by 3 days recovery. (D) Body weights shown as percentage of starting weight (n = 5 to 9 per group). (E) Colon length. (F) and (G) Expression of indicated genes in distal colon tissue, normalized to *Gapdh* and shown as relative mean of control group. (H–K) Trained and untrained male mice received liposome or PBS injection. Then all mice were infected with *C. rodentium* by oral gavage, and outcomes were analyzed on day 14 after infection. (H) Body weights shown as percentage of starting weight (n = 7 to 9 per group). (I) Colon length. (J) and (K) Expression of indicated genes in distal colon tissue, normalized to *Gapdh* and shown as relative mean of control group. Data points represent individual mice, pooled from two independent experiments except for (C). All data are represented as means ± SEM. p values were calculated by one-way ANOVA or Student’s *t* test for (B) and (D–K). For (B), (D), and (H), Student’s *t* test was performed independently at each time point; ∗p < 0.05, ∗∗p < 0.01, and ∗∗∗p < 0.001.

Macrophage, the most well-studied innate immune cell in trained immunity, are critical for maintaining intestinal homeostasis and for the continuous renewal of intestinal epithelial cells and mucus in the intestinal tract.^28^ Therefore, we questioned how macrophage changes in gut and draining LNs after WD feeding and its potential role in WD training. The requirement for macrophages in WD trained colitis protection was tested by injecting clodronate liposomes to deplete macrophages during WD period.^29^ Control mice received blank liposome and all mice were challenged with DSS or infected with *C. rodentium* after training (Figure 7A). Clodronate administration resulted in reduced weight gain, but these mice recovered (Figure 7B) and had similar colon length (Figure 7C) compared to the control liposome group by the end of the rest period. Following DSS or *C. rodentium* challenge, trained mice with blank liposome injection had better colitis outcomes than untrained mice with PBS injection as before. However, clodronate liposome injection reversed the protective effects on colitis severity and intestinal barrier phenotypes in trained mice (Figures 7D–7K). Together, these data pinpoint WD trained macrophages as a major mediator of the tissue protective effect in our model.

### Mevalonate pathway and macrophage training dependent protective effect on colitis is shared by female mice

**Figure 8:**
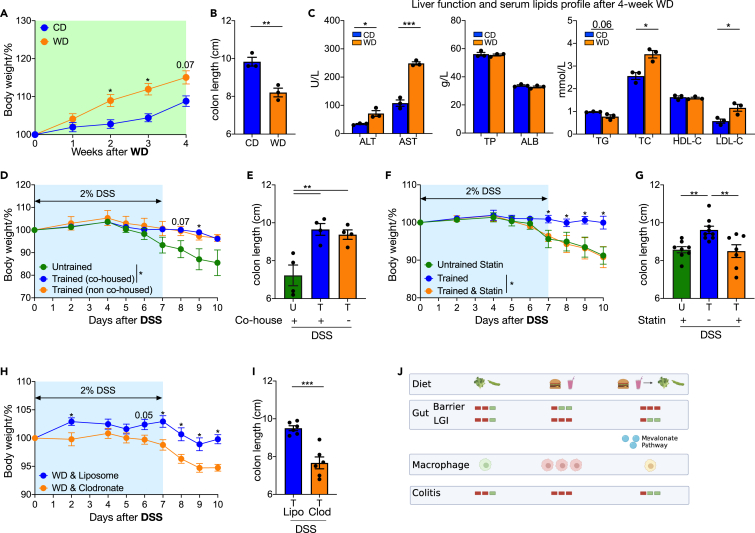
Mevalonate pathway and macrophage training dependent protective effect on colitis is shared by female mice (A–C) WT female mice were feed on WD and outcomes were analyzed 4 weeks later. (A) Body weights shown as percentage of starting weight (n = 7 to 23 per group). (B) Colon length. (C) Serum ALT, AST, TP, ALB, TG, TC, HDL-C and LDL-C level. (D–E) WT female mice were trained by 4-week ‘on and off’ WD, a control group received normal chow diet and drinking water. Then 2% DSS in drinking water were administered for 7 days to induce inflammation followed by 3 days’ recovery. (D) Body weights shown as percentage of starting weight (n = 4 per group). (E) Colon length. (F and G) Trained and untrained female mice received Fluvastatin whole training and rest period. Then 2% DSS in drinking water were administered for 7 days to induce inflammation followed by 3 days recovery. (F) Body weights shown as percentage of starting weight (n = 8 to 9 per group). (G) Colon length. (H and I) Trained and untrained female mice received liposome injection. Then 2% DSS in drinking water were administered for 7 days to induce inflammation followed by 3 days recovery. (H) Body weights shown as percentage of starting weight (n = 5 to 6 per group). (I) Colon length. (J) Graphical summary. Data points represent individual mice, pooled from two independent experiments except for (B–E) from one experiment. All data are represented as means ± SEM. p values were calculated by Student’s *t* test or one-way ANOVA for (E) and (G). For (A), (D), (F), and (H), Student’s *t* test was performed independently at each time point; ∗p < 0.05, ∗∗p < 0.01, and ∗∗∗p < 0.001.

Gender differences have been ignored for centuries, resulting in a data gap. Recent papers have shown sex makes a large difference on the pathophysiology IBD and NASH.^30^ Our data thus far were performed in male mice, which are more susceptible to WD induced effects. In addition, estrogens are thought to protect female mice from DSS colitis.^31^ We therefore tested whether the WD training model operates in female mice. Consistent with published reports,^30^ female mice gained less weight under the WD feeding regime (Figure 8A). Nevertheless, colon shortening (Figure 8B), hepatocyte injury and serum cholesterol elevation (Figure 8C) were all mimicked in female mice. WD trained mice had reduced weight loss and longer colons then untrained mice in DSS induced colitis model, and these effects were again independent of co-housing during chow diet rest (Figures 8D and 8E). Furthermore, the protective effect was totally abrogated if statin was administered (Figures 8F and 8G) or macrophages were depleted (Figures 8H and 8I). Thus, we identified upregulation of the mevalonate pathway and macrophages as essential regulators of WD training induced colitis protection in both male and female mice (Figure 8J, generated by Biorender).

## Discussion

Diet links environmental and human health. Inadequate intake of essential fatty acids, minerals, vitamins and proteins are a health threat to the world’s poorest people. In contrast, excess consumption of fats and sugars resulting from the so-called WD are also associated with chronic diseases, including type 2 diabetes, cancer and coronary heart disease. Increased WD consumption is also associated with increased intestinal inflammatory disease.^32^ Disturbance in gut barrier, inflammatory balance, gut flora and serum lipids were confirmed among both male and female mice after WD feeding in our study, all of which could exacerbate colitis severity. WD feeding induced changes that were even observed after 24-h feeding,^33^ and 4-week, 8-week, and 16-week feeding mice shared most gut phenotypes, suggesting the diet itself matters rather than duration. On the other hand, WD accompanied changes could be restored after transferring to a normal diet. Christ et al. found 4-week WD elevated growth factors, cytokines, chemokines and acute phase proteins in *Ldlr*^*−/−*^ mice circulation, all of which returned to baseline level after 4-week chow diet recovery.^16^ Our data also showed that WD-induced changes in macrophage markers came back to basal level after a period of normal chow diet. However, expression of colon cytokines including IL-10 suggested that resolution of WD-induced colon inflammation after return to a healthier diet led to an enhanced anti-inflammatory milieu. Furthermore, the deficits in gut barrier observed during WD consumption were replaced by increased mucus and expression of barrier function genes on shifting mice back to the chow diet. Naik et al. reported that previous inflammation sensitized skin epithelial stem cells responsiveness to subsequent stressors leading to enhanced wound healing.^34^ Intestinal epithelial cells replenishment, including goblet cell and epithelium cell, rely on the proliferation and differentiation of Lgr5^+^ intestinal stem cells (ISCs),^35^ so it will be also interesting to determine diets training effects on ISCs in future study.

The intestinal surface is constantly exposed to external environmental particles and microbes, but integrated gut barrier under physiological condition limits the penetration of those disease-causing agents. Although the pathogenesis of IBD is complex and incompletely understood, it is well established that gut barrier prevents inappropriate activation of the immune system.^36^ Increased intestinal permeability occurs before the onset of gut inflammation and is associated with later development and long-term outcomes of IBD.^17^^,^^18^ Thus, gut barrier reconstruction is now being raised as a critical point in IBD therapy.^37^ Being an inflammatory cytokines, IL-17 signaling actively participant in maintaining intestinal integrity through ISCs self-regeneration and differentiation into secretory cells.^38^ After training, we found more goblet cell and higher its specific genes expression which may be related with elevated *Il17a* expression by 4-week WD feeding. Notably, *Il17a* expression decreased in 16-week WD mice, which may indicate a possible different outcome between short-term and long-term WD training.

Gut microbiota and their metabolites affect gut barrier function. For instance, *Akkermansia muciniphila* is well known as a mucin-degrading bacteria by utilizing the mucin glycan for their maintenance,^39^ but at the same time *A. muciniphila*-derived extracellular vesicles increase tight junction proteins expression and protected mice form DSS-induced colitis.^40^ Similar to previous findings,^41^ we observed a decrease of *A. muciniphila* in WD mice compared with control mice. However, the abundance of *A. muciniphila* was low in both trained and untrained mice. In fact, the only enriched bacterial species in trained mice compared to control mice was *H. typhlonius,* which is thought to cause irritable bowel syndrome,^42^ intestinal tumorigenesis,^43^ and even typhlocolitis,^44^ in contrast to the protection that we observed. This suggests a complex interaction of microbiota with gut barrier in our model. *Faecalibaculum rodentium* was enriched in WD mice. Although *F. rodentium* has been shown to promote epithelial proliferation and turnover^45^ and protect from intestinal tumorigenesis,^46^ *Kawano* et al. demonstrated that dietary sugar increases *F. rodentium,* displacing Th17-inducing segmented filamentous bacteria (SFB) and increasing the risk for metabolic disease.^47^ Overall, the differences between co-housed trained and untrained mice were small, and do not immediately correlate with the functional outcomes during colitis challenge. In humans, changes in diet, living arrangements, and the use of medications (especially antibiotics) may trigger microbial community changes, but the microbial composition is thought to recover once such challenges are removed and have been found to remain relatively stable throughout life span.^48^^,^^49^ It is worth noting that the microbiota composition of co-housed untrained mice mimicked the trained mice, and both showed changes compared to mice maintained on chow diet without co-housing. However, these co-housed untrained mice showed no protective benefit compared to controls that were not co-housed, suggesting a limited role of gut microbiota changes in training benefits.

Traditionally, immunological memory is considered in context of adaptive immune response in mammals. However, this concept is challenged by studies from invertebrates^50^ as well as some new findings in the mammalian innate immune system.^51^^,^^52^ Cells of the innate immune system, like macrophages, show an altered response to secondary stimulus, which has been termed innate immune training.^51^^,^^52^^,^^53^ The concept of innate immune training was first raised by randomized clinical trials and observational studies of pediatric vaccinations that induced non-specific beneficial effects on mortality from other diseases.^54^^,^^55^ Since then, it has become clear that innate immune training applies not only to innate immune cells such as macrophages but also to stromal and epithelial cells.^34^^,^^56^^,^^57^ Inducers of immune training have also been defined and include BCG, β-glucan, LPS as well as low-dose DSS.^15^^,^^16^ Mechanistically, epigenetic modification^27^ and metabolic re-programming including changes in glycolysis,^58^ glutaminolysis,^59^ as well as cholesterol synthesis mevalonate pathway^25^ are strongly associate with trained immunity establishment. Moreover, cytokines, like IL-1β and IL-17A, are involved.^15^^,^^16^ Our data confirmed that mevalonate-dependent pathways acted as key mediators for WD induced cross protection on colitis. Statin, a mevalonate pathway inhibitor, is widely used in clinic to prevent cardiovascular events. In colitis model, conflicting results have been reported on the influence of statins, especially on DSS induced colitis.^60^^,^^61^ Findings in our experiment show that statin blocked protective training effects. This may explain reported statin adverse effects like statin-induced colitis considering introduction of trained immunity by endogenous stimuli under physiological conditions.^62^ What’s more, WD training protection is independent of hypercholesterolemia induction, as short-term statin treatment only during WD feeding made no difference to the training outcome, indicating that cells required ongoing changes in mevalonate activity, as has been reported for trained myeloid cells. Another interesting finding of the statin study was that it did not alter weight gain for chow diet mice, but prevented high fat and high carbohydrate diet induced obesity, giving the potential application of statin to metabolic disease treatment.

Both preclinical and clinical data demonstrate different outcome of female and male in IBD and NASH.^30^^,^^31^ Besides, recently studies also demonstrated that trained immunity had sexual difference.^63^ Administration of the live attenuated measles-mumps-rubella (MMR) vaccine only shown a protective effect against SARS-CoV-2 disease in males but not females^64^ and BCG vaccination also resulted in significant reductions in inflammatory proteins in males health volunteer only.^65^ Although the severity of colitis was different between sexes in our murine study, a common and broad protection effect of WD training on colitis was found in both sexes, suggesting the benefits of healthy diet on gut inflammation are not gender-specific.

In conclusion, the findings from this study confirm short-term inflammatory training by WD results in a non-specific protection on future colitis. Furthermore, the cholesterol synthesis pathway is essential for inducing protective effect in a macrophage dependent manner. Clinical guidelines agree the idea that proper diet control is an important factor in regulating IBD relapse. Exclusive enteral nutrition (EEN), which involves the exclusive use of a liquid diet, is used as standard therapy to induce mucosal healing in children IBD.^66^ Perhaps more surprisingly, our data suggest that brief periods of WD consumption may even be beneficial to gut health if followed by a return to a carefully balanced nutritious diet. For many mammals, the drive to consume high calorie foods is important for survival and periods of feasting to take advantage of food abundance are followed by periods of more meager food intake. Humans followed this pattern before the advent of farming and every-increasing availability of affordable high-fat, high-sugar food products. It is interesting to speculate that the intestine has evolved mechanisms to cope with the transient onslaught of these high calorie foods, using these periods to bolster intestinal defenses and immune activation to better cope with insults that may occur in more nutrient-lean times when less energy is available to commit to host defense.

### Limitations of the study

Several limitations need to be considered. First, we identified important roles of macrophages and innate myeloid training, but the exact epigenetic changes and metabolic re-programming will require further study. Second, the possible influence of WD on enterocytes and stem cell will be interesting to explore in our model.

## STAR★Methods

### Key resources table

**Table:** 

REAGENT or RESOURCE	SOURCE	IDENTIFIER
**Antibodies**		

Rabbit polyclonal anti-MUC2	Abcam	ab90007
Mouse monoclonal anti-ECAD (clone 4A2)	Abcam	ab231303

**Bacterial and virus strains**		

*Citrobacter rodentium* strain DBS100	ATCC	ATCC 51459

**Critical commercial assays**		

Western diet	Teklad	TD. 120528
DSS (36,000-50,000 M.Wt),	MP Biomedicals	160110
Fluvastatin	Shanghai Bide Pharmatech Ltd.	BD23193
Liposome-encapsulated clodronate	LIPOSOMA	https://clodronateliposomes.com/
Lipopolysaccharides,≥98%	Solarbio Life Sciences	Cat#L8880
Palmitic acid,BioXtra,≥99.0%	Sigma-Aldrich	Cat#P5585-10G
Dulbecco's modified eagle medium	Gibco	Cat#C11995500BT
EasyPure Stool Genomic DNA Kit	TransGen Biotech	EE101
E.Z.N.A. Total RNA Kit II RNA Isolation Kit	OMEGA	SKU: R6934
PerfectStart Uni RT-qPCR Kit	Transgen	AUQ-01

**Deposited data**		

RNA Seq data of colon tissue	Genome Sequence Archive	GAS: CRA017088

**Experimental models: Cell lines**		

Mouse: RAW264.7 cells	ATCC	TIB-71

**Experimental models: Organisms/strains**		

Mouse: C57BL/6 mice	Hunan SJA Laboratory Animal Co., Ltd	N/A

**Oligonucleotides**		

Primers for RT-PCR, see Table S1	This paper	N/A

**Software and algorithms**		

GraphPad Prism	Graphpad Software	https://www.graphpad.com
R	v4.1.1	cran.r-project.org

### Resource availability

#### Lead contact

Further information and requests for resources and reagents should be directed to and will be fulfilled by the Lead Contact, Xiaowei Liu (liuxw@csu.edu.cn).

#### Materials availability

This study did not generate new unique reagents.

### Experimental model and subject details

#### Mice

C57BL/6 mice was obtained from the Hunan SJA Laboratory Animal Co., Ltd (Changsha, China). All experiments included age- and sex-matched littermate controls, and both males and females aged between 7 and 8 weeks were used. Mice were housed under specific pathogen-free conditions in Central South University. Protocols were approved by Institutional Ethics Committee for animal procedures of the Central South University (No. CSU-2022-0082). For chemical induced colitis, mice received 1% or 2% indicated DSS in their drinking water for 7 days, followed by 3 days of distilled water without DSS. For infection induced colitis, mice were orally gavaged with fresh cultured 10^9^ colony-forming units (CFUs) of *C. rodentium* strain DBS100. For *in vitro* study, American Type Culture Collection TIB-71 cell line RAW 264.7 was used.

### Method details

#### Cell and treatments

RAW 264.7 were obtained American Type Culture Collection TIB-71. Cells were stimulated *in vitro* with palmitic acid (PA, 250μM, Sigma-Aldrich) for 6 hr. After 6-day rest, cells were restimulated with LPS (10 ng/ml, Solarbio Life Sciences). After 24h cells were collected.

#### Diets

Male and female C57BL/6J mice were fed a Western diet (Teklad TD. 120528) consisting of a forage with 17.3% protein, 21.2% fat and 48.5% carbohydrates and drinking water with glucose (18.9 g/L) and fructose (23.1 g/L) for 4, 8 or 16 weeks, or a control chow diet (MD17121, Research Diets) with distilled normal drinking water. To wash out WD effects, mice were subsequently subjected to regular chow diet for additional 4 weeks after 4-week WD feeding.

#### DSS-induced colitis

Mice received 1% or 2% indicated DSS (36,000 to 50,000 molecular weight; MP Biomedicals) in their drinking water for 7 days, followed by 3 days of distilled water without DSS. Control animals received distilled water for the entire period. Mice were monitored at D2 and every day from D4 for body weight until D10.

#### *C. rodentium* infection and fecal bacteria detection

Mice were orally gavaged with fresh cultured 10^9^ colony-forming units (CFUs) of *C. rodentium* strain DBS100 (American Type Culture Collection 51459). Body weight was measured when collecting fresh feces every four days. For fecal *C. rodentium* polymerase chain reaction (PCR), DNA in feces was isolated with the EasyPure Stool Genomic DNA Kit (TransGen Biotech), Espb gene expression was quantified by quantitative PCR (qPCR) and normalized relative to total bacterial 16S rRNA.

#### Fluvastatin treatment

10 mg/kg per day Fluvastatin^67^ (from Shanghai Bide Pharmatech Ltd., BD23193, 98%) was administered daily in the drinking water for 4 weeks or 8 weeks. The daily dose of the drug was adjusted according to the volume of drunk water and the body weight (measured every week).

#### Macrophage deletion

Liposome-encapsulated clodronate or PBS was purchased from LIPOSOMA (https://clodronateliposomes.com/). Depletion of macrophages was achieved by injection of 0.2 ml of liposome-encapsulated clodronate i.p. twice a week for 4 weeks (liposome-encapsulated PBS as control), according to the provider's instructions. Mice weight was obtained before every injection.

#### Quantitative PCR

RNA was isolated with the E.Z.N.A. Total RNA Kit II RNA Isolation Kit (OMEGA), and cDNA was generated with *PerfectStart* Uni RT-qPCR Kit (Transgen), followed by real-time PCR (RT-PCR) using SYBR Green Master mix with ROX and ABI QuantStudio 7 Flex instrument. RT qPCR primers sequence was shown in Table S1. Gene expression was normalized to *Gapdh*.

#### Histology

Distal colon was fixed with 4% paraformaldehyde and embedded in Paraffin. Tissue sections (5 μm) were prepared, deparaffinized, and stained with hematoxylin and eosin. Histological scores were assigned by experimenters “blinded” to sample identity. Colonic epithelial damage was scored according to previous publication.^68^ For PAS staining, tissue was stored in Carnoy's solution. After dewaxing, sections were stained with PAS dye solution B for 10-15 min, PAS dye solution A for 25-30 min in the dark and PAS dye solution C for 30s. Primary antibodies used for immunofluorescence staining were MUC2 (Abcam) and ECAD (Abcam). All images were acquired with *Leica* microscope.

#### Liver function and serum lipids analysis

Liver function readout (including alanine aminotransferase (ALT) and aspartate aminotransferase (AST), total protein (TP) and albumin) as well as serum lipids (including total triglycerides (TG), total cholesterol (TC), high-density lipoprotein cholesterol (HDL-C) and low-density lipoprotein cholesterol (LDL-C) were analyzed by AU680 autoanalyser (Beckman Coulter, Brea, USA).

#### 16S rRNA gene amplicon sequencing and analysis

Total genomic DNA samples were extracted using the OMEGA Soil DNA Kit (M5635-02) (Omega Bio-Tek, Norcross, GA, USA). PCR amplification of the bacterial 16S rRNA genes V3–V4 region was performed using the forward primer 338F (5'-ACTCCTACGGGAGGCAGCA-3') and the reverse primer 806R (5'-GGACTACHVGGGTWTCTAAT-3'). Sample-specific 7-bp barcodes were incorporated into the primers for multiplex sequencing. PCR amplicons were purified with Vazyme VAHTSTM DNA Clean Beads (Vazyme, Nanjing, China) and quantified using the Quant-iT PicoGreen dsDNA Assay Kit (Invitrogen, Carlsbad, CA, USA). After the individual quantification step, amplicons were pooled in equal amounts, and pair-end 2x250 bp sequencing was performed using the Illumina NovaSeq platform with NovaSeq 6000 SP Reagent Kit (500 cycles) at Suzhou PANOMIX Biomedical Tech Co. LTD. Microbiome bioinformatics were performed with QIIME2 2019.4^69^ with slight modification according to the official tutorials (https://docs.qiime2.org/2019.4/tutorials/). Alpha-diversity metrics (*Shannon* and *Simpson*) and beta diversity metrics (weighted UniFrac and unweighted UniFrac) were estimated. Bray-Curtis metrics and UniFrac distance metrics were visualized via principal coordinate analysis (PCoA), nonmetric multidimensional scaling (NMDS) and unweighted pair-group method with arithmetic means (UPGMA) hierarchical clustering. Principal component analysis (PCA) was also conducted based on the genus-level compositional profiles. The significance of differentiation of microbiota structure among groups was assessed by ANOSIM (Analysis of similarities). LEfSe (Linear discriminant analysis effect size) was performed to detect differentially abundant taxa across groups using the default parameters.

#### RNA-seq library preparation and sequencing

Total RNA was extracted from the tissues using TRIzol® Reagent according the manufacturer’s instructions (Magen). Paired-end libraries were prepared using a ABclonal mRNA-seq Lib Prep Kit (ABclonal, China) following the manufacturer’s instructions. The mRNA was purified from 1 μg total RNA using oligo (dT) magnetic beads followed by fragmentation carried out using divalent cations at elevated temperatures in ABclonal First Strand Synthesis Reaction Buffer. Subsequently, first-strand cDNAs were synthesized with random hexamer primers and Reverse Transcriptase (RNase H) using mRNA fragments as templates, followed by second-strand cDNA synthesis using DNA polymerase I, RNAseH, buffer, and dNTPs. The synthesized double stranded cDNA fragments were then adapter- ligated for preparation of the paired-end library. Adaptor-ligated cDNA were used for PCR amplification. PCR products were purified (AMPure XP system) and library quality was assessed on an Agilent Bioanalyzer 4150 system. Finally, the library preparations were sequenced on MGISEQ-T7 and 150 bp paired-end reads were generated. The data generated from BGI platform were used for bioinformatics analysis. All of the analyses were performed using an in-house pipeline from Shanghai Applied Protein Technology. The major software and parameters are as follows.

### Quantification and statistical analysis

The statistical test method can be found in the figure legends. Experimental results were analyzed for significance using one-way analysis of variance (ANOVA) with Tukey’s multiple comparisons test (for multiple groups) or unpaired *Student’s t* test. Statistical analyses were performed using GraphPad Prism. P values are shown as ∗P < 0.05, ∗∗P < 0.01, ∗∗∗P < 0.001, and ∗∗∗∗P < 0.0001 where statistical significance was found, and all data are represented as means ± SEM. Individual points in graphs represent biological replicates (i.e., individual mice) pooled from multiple experiments.

## Data Availability

• RNA-seq data have been deposited at NGDC OMIX and are publicly available as of the date of publication. Accession numbers are listed in the key resources table. • No original code is generated in this paper. • Any additional information required to reanalyze the data reported in this paper is available from the lead contact upon request.
