# Supplementary material for: Temporary consumption of western diet trains the immune system to reduce future gut inflammation
Source: iScience. 2023 May 20;26(6):106915. doi: 10.1016/j.isci.2023.106915 (PMC10250831; doi:10.1016/j.isci.2023.106915)
Supplement: Document S1. Figures S1–S8 and Table S1 [file mmc1.pdf]

iScience, Volume *26*

Supplemental information

Temporary consumption of western diet trains the immune system to reduce future gut inflammation

Dongwen Wu, Xiaotong Wang, Xiang Yang, Lei Gu, Mandy J. McGeachy, and Xiaowei Liu

Supplemental Table

Table S1 Primer sequences of RT-PCR, related to STAR Methods

Gene	Forward Sequence	Reverse Sequence
16S rRNA	ATTACCGCGGCTGCTGGC	ACTCCTACGGGAGGCAGCAGT
Agr2	GCAGTTTGTTCTCCTCAACCTGG	GTATCGTCCAGTGATGTCTGCC
Cd163	GGCTAGACGAAGTCATCTGCAC	CTTCGTTGGTCAGCCTCAGAGA
Cd19	GCCACAGCTTTAGATGAAGGCAC	CATCCACCAGTTCTCAACAGCC
Cd206	GTTCACCTGGAGTGATGGTTCTC	AGGACATGCCAGGGTCACCTTT
Cd3e	GCTCCAGGATTTCTCGGAAGTC	ATGGCTACTGCTGTCAGGTCCA
Clca1	CTGCCGCTAAAGAGCTTGAGCA	ATCGCCGCATTTCCTGAGGAGA
Ctsz	GTGTCAGCAACGATGGCATCGA	CCTTGTAGGTGCTGGTCACGAT
Espb	ATGCCGCAGATGAGACAGTTG	CGTCAGCAGCCTTTTCAGCTA
Fcgbp	CCATCCACAAGAATGAGATCGGC	GTCAGTCTCCAGCACAGCCTTT
Foxp3	CCTGGTTGTGAGAAGGTCTTCG	TGCTCCAGAGACTGCACCACTT
Gata3	TCATTAAGCCCAAGCGAAGG	GTCCCCATTGGCATTCCTC
Hmgcr	GCTCGTCTACAGAAACTCCACG	GCTTCAGCAGTGCTTTCTCCGT
Hmgcs	GGAAATGCCAGACCTACAGGTG	TACTCGGAGAGCATGTCAGGCT
Il10	CCAAGCCTTATCGGAAATGA	TTTTCACAGGGGAGAAATCG
Il13	AACGGCAGCATGGTATGGAGTG	TGGGTCCTGTAGATGGCATTGC
Il17a	GCCCTCAGACTACCTCAACC	ACACCCACCAGCATCTTCTC
Il18	GACAGCCTGTGTTCGAGGATATG	TGTTCTTACAGGAGAGGGTAGAC
Il1b	GAGAGCCGGGTGACAGTATC	TGACAAACTTCTGCCTGACG
Il22	GCTTGAGGTGTCCAACTTCCAG	ACTCCTCGGAACAGTTTCTCCC
Il23a	GGTGGCTCAGGGAAATGT	GACAGAGCAGGCAGGTACAG
Il6	AGTTGCCTTCTTGGGACTGA	CAGAATTGCCATTGCACAAC
iNOS	GTTCTCAGCCCAACAATACAAGA	GTGGACGGGTCGATGTCAC
Irg1	GTTCCTGGGAACCACTACG	GATGTCTGGCTGACCCCAAA
Ldlr	GAATCTACTGGTCCGACCTGTC	CTGTCCAGTAGATGTTGCGGTG
Muc2	GCTGACGAGTGGTTGGTGAATG	GATGAGGTGGCAGACAGGAGAC
Mvd	CAGCTAGTCCACCGCTTCAACA	CAAACTCAGCCACAGTGTCCTC
Mvk	ATGCTTCAGCGACTGGACACGA	AGCAGAGCCATGCCTTCATTGC
Nrf2	CAGCATAGAGCAGGACATGGAG	GAACAGCGGTAGTATCAGCCAG
Ocln	CCTCCAATGGCAAAGTGAAT	CTCCCCACCTGTCGTGTAGT
Pmvk	ACTTCGTGACCGAGAGGCTGAA	CTTGTAGGTGCTCGCATCCAGA
Reg3b	TGGCTCCTACTGCTATGCCTTG	CGCTATTGAGCACAGATACGAGG
Reg3g	CGTGCCTATGGCTCCTATTGCT	TTCAGCGCCACTGAGCACAGAC
Rorc	GCAGCGCTCCAACATCTTCT	ACGTACTGAATGGCCTCGGT
Sdha	GAGATACGCACCTGTTGCCAAG	GGTAGACGTGATCTTTCTCAGGG
Spdef	CACGTTGGATGAGCACTCGCTA	AGCCACTTCTGCACGTTACCAG
Sqle	TGTTGCGGATGGACTCTTCTCC	GTTGACCAGAACAAGCTCCGCA
Stat1	GCCTCTCATTGTCACCGAAGAAC	TGGCTGACGTTGGAGATCACCA
Stat3	AGGAGTCTAACAACGGCAGCCT	GTGGTACACCTCAGTCTCGAAG

Tbet	CGGCTGCATATCGTTGAGGT	GTCCCCATTGGCATTCCTC
Tff1	CAGGCCCAGGCCCAGGAAGA	CTGTCATCAAAACAGCAACCTCTC
Tnfa	CGTCAGCCGATTTGCTATCT	CGGACTCCGCAAAGTCTAAG
Zg16	CCAGGACATCCTCTTACAGTGG	TCACTCCACACTGTGCCATAGC
Zo1	GCCGCTAAGAGCACAGCAA	GCCCTCCTTTTAACACATCAGA

Supplemental Figure

Figure S1 16-week WD induces low-grade inflammation and skewed cytokines profiles, related to Figure 1

WT male mice were fed on WD and outcomes were analyzed 16 weeks later.
(A), (E), (F) and (G) Expression of indicated genes in distal colon tissue, normalized to Gapdh and shown as relative mean of control group.
(B) representative images of H&E of tissue sections from distal colon.
(C) 16s rRNA level in MLNs.
(D) Colon length.
(H) Expression of indicated genes in MLN, normalized to Gapdh and shown as relative mean of control group.
Data points represent individual mice and were from one experiment. All data are represented as means ± SEM. *P* values were calculated by Student's *t* test; *P < 0.05, **P < 0.01, and***P < 0.001.

Figure S2 WD training had no effects on future WD challenge, related to Figure 3

WT male mice were trained by 4-week 'on and off' WD, a control group received normal chow diet and drinking water. Then all mice were fed on WD and outcomes were analyzed 4 weeks later.
(A) Experimental design.
(B) Body weights (n = 5 to 6 per group).
(C) Body weights shown as percentage of starting weight (n = 5 to 6 per group).
(D) Colon length.
(E) and (F) Expression of indicated genes in distal colon tissue, normalized to Gapdh and shown as relative mean of control group.
Data points represent individual mice and were from two independent experiment. All data are represented as means ± SEM. *P* values were calculated by Student's *t* test; *P < 0.05, **P < 0.01, and***P < 0.001.

Figure S3 WD training protection effect is independent on chow diet mice co-housing, related to Figure 4

(A) to (D) WT male mice were trained by 4-week 'on and off' WD, a control group received normal chow diet and drinking water. Then 2% DSS in drinking water were administered for 7 days to induce inflammation followed by 3 days recovery.
(A) Experimental design.
(B) Body weights shown as percentage of starting weight (n = 4 to 5 per group).
(C) Colon length.
(D) Expression of indicated genes in distal colon tissue, normalized to *Gapdh* and shown as relative mean of control group.

Data points represent individual mice and were from one experiment. All data are represented as means ± SEM. *P* values were calculated by Student's *t* test; *P < 0.05, **P < 0.01, and***P < 0.001.

(E) and (F) Fecal samples were collected from chow diet (4 mice), 8-week WD (5 mice), untrained (6 mice) and trained mice (6 mice) and did 16s rRNA sequencing.

(E) Shannon Diversity Index for Alpha diversity.

(F) NMDS plot for Beta diversity.

P values were calculated by Turkey test; *P < 0.05, **P < 0.01, and***P < 0.001.

Figure S4 Serum lipids profile and 4-week stain treatment effects on body weight and colitis, related to Figure 5

(A) WT male mice were feed on WD and outcomes were analyzed 8 weeks later. Serum alanine aminotransferase (ALT), aspartate aminotransferase (AST), total protein (TP), albumin (ALB), total glyceride (TG), total cholesterol (TC), high density lipoprotein cholesterol (HDL-C) and low density lipoprotein cholesterol (LDL-C) level.

(B) Mevalonate pathway and its key enzymes.

(C to E) WD or chow diet male mice received Fluvastatin during training period and outcomes were analyzed 4 weeks later.

(C) Experimental design.

(D) Body weights shown as percentage of starting weight (n = 3 per group).

(E) Colon length.

Data points represent individual mice and were from one experiment. All data are represented as means ± SEM. *P* values were calculated by Student's *t* test or one-way ANOVA; *P < 0.05, **P < 0.01, and***P < 0.001.

Figure S5 4-week statin treatment did not reverse WD training effects on DSS induced colitis, related to Figure 5

Trained male mice received 4-week Fluvastatin. Then 2% DSS in drinking water were administered for 7 days to induce inflammation followed by 3 days recovery.

(A) Experimental design.

(B) Body weights shown as percentage of starting weight (n = 5 per group).

(C) Colon length.

(D) and (E) Expression of indicated genes in distal colon tissue, normalized to *Gapdh* and shown as relative mean of control group.

Data points represent individual mice and were from one experiment. All data are represented as means ± SEM. *P* values were calculated by Student's *t* test; *P < 0.05, **P < 0.01, and***P < 0.001.

Figure S6 4-week statin treatment did not reverse WD training effects on C. rodentium infection induced colitis, related to Figure 5

Trained male mice received 4-week Fluvastatin. All mice were infected with *C. rodentium* and outcomes were analyzed on day 14 after infection.

(A) Experimental design.

(B) Body weights shown as percentage of starting weight (n = 5 per group).
(C) Colon length.
(D) and (E) Expression of indicated genes in distal colon tissue, normalized to *Gapdh* and shown as relative mean of control group.
Data points represent individual mice and were from one experiment. All data are represented as means ± SEM. *P* values were calculated by Student's *t* test; *P < 0.05, **P < 0.01, and***P < 0.001.

Figure S7 8-week statin treatment effects on gut barrier and colon inflammation, related to Figure 5

Trained and untrained male mice received Fluvastatin whole training and rest period and outcomes were analyzed after chow diet rest.
(A) and (B) Expression of indicated genes in distal colon tissue, normalized to Gapdh and shown as relative mean of control group.
(C) representative images of PAS staining of tissue sections from distal colon.
Data points represent individual mice and were from two independent experiment. All data are represented as means ± SEM. *P* values were calculated by Student's *t* test; *P < 0.05, **P < 0.01, and***P < 0.001.

Figure S8 WD changed macrophage polarization, related to Figure 6

(A) and (B) WT male mice were feed on WD and outcomes were analyzed 16 weeks later. Expression of indicated genes in distal colon tissue and MLN, normalized to *Gapdh* and shown as relative mean of control group.
(C) and (D) WT male mice were feed on WD and outcomes were analyzed 4 weeks later. Expression of indicated genes in distal colon tissue and MLN, normalized to *Gapdh* and shown as relative mean of control group.
(E) WT male mice were trained by 4-week 'on and off' WD. Expression of indicated genes in distal colon tissue and MLN, normalized to *Gapdh* and shown as relative mean of control group.
Data points represent individual mice and were from two independent experiments. All data are represented as means ± SEM. *P* values were calculated by Student's *t* test; *P < 0.05, **P < 0.01, and***P < 0.001.

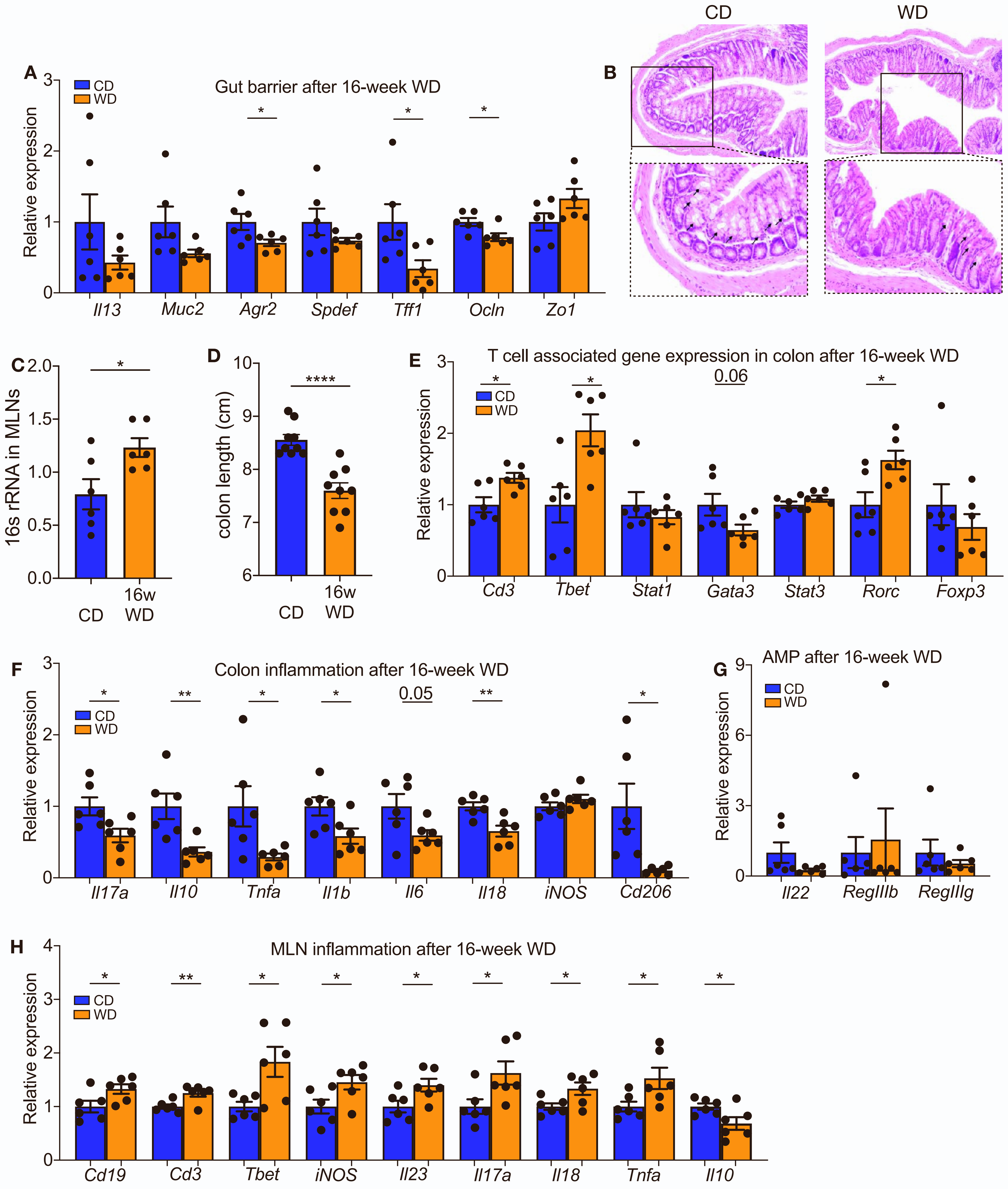
A
Gut barrier after 16-week WD
CD
WD
Relative expression
Il13
Muc2
Agr2
Spdef
Tff1
Ocln
Zo1
B
CD
WD
C
16s rRNA in MLNs
CD
16w WD
D
colon length (cm)
CD
16w WD
E
T cell associated gene expression in colon after 16-week WD
0.06
Relative expression
Cd3
Tbet
Stat1
Gata3
Stat3
Rorc
Foxp3
F
Colon inflammation after 16-week WD
0.05
Relative expression
Il17a
Il10
Tnfa
Il1b
Il6
Il18
iNOS
Cd206
G
AMP after 16-week WD
Relative expression
Il22
RegIIIb
RegIIIg
H
MLN inflammation after 16-week WD
Relative expression
Cd19
Cd3
Tbet
iNOS
Il23
Il17a
Il18
Tnfa
Il10

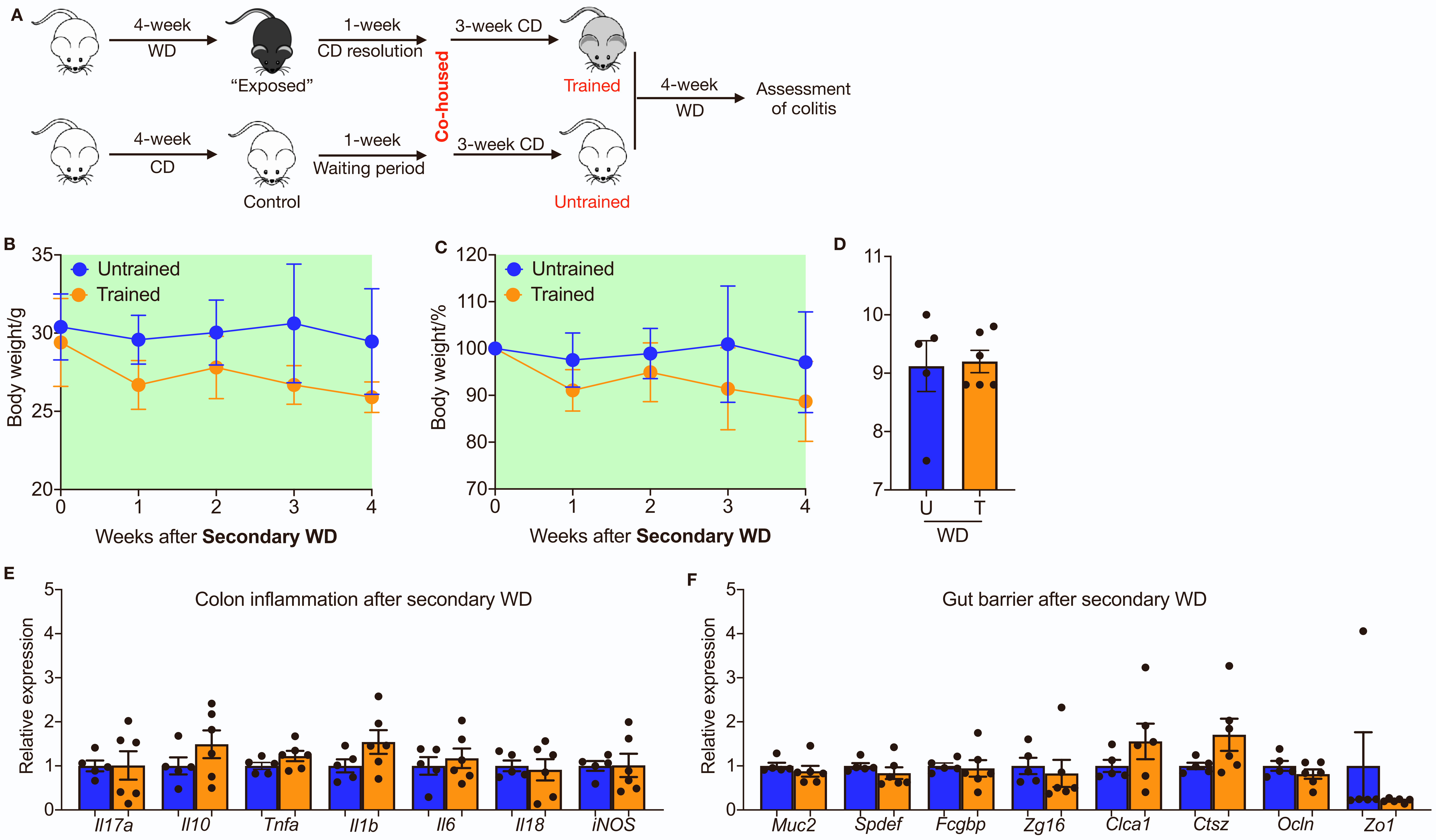

A
4-week
WD
"Exposed"
1-week
CD resolution
3-week CD
Trained
Co-housed
4-week
WD
Assessment
of colitis
4-week
CD
Control
1-week
Waiting period
3-week CD
Untrained
B
Untrained
Trained
Body weight/g
Weeks after Secondary WD
C
Untrained
Trained
Body weight/%
Weeks after Secondary WD
D
U
T
WD
E
Colon inflammation after secondary WD
Relative expression
Il17a
Il10
Tnfa
Il1b
Il6
Il18
iNOS
F
Gut barrier after secondary WD
Relative expression
Muc2
Spdef
Fcgbp
Zg16
Clca1
Ctsz
Ocln
Zo1

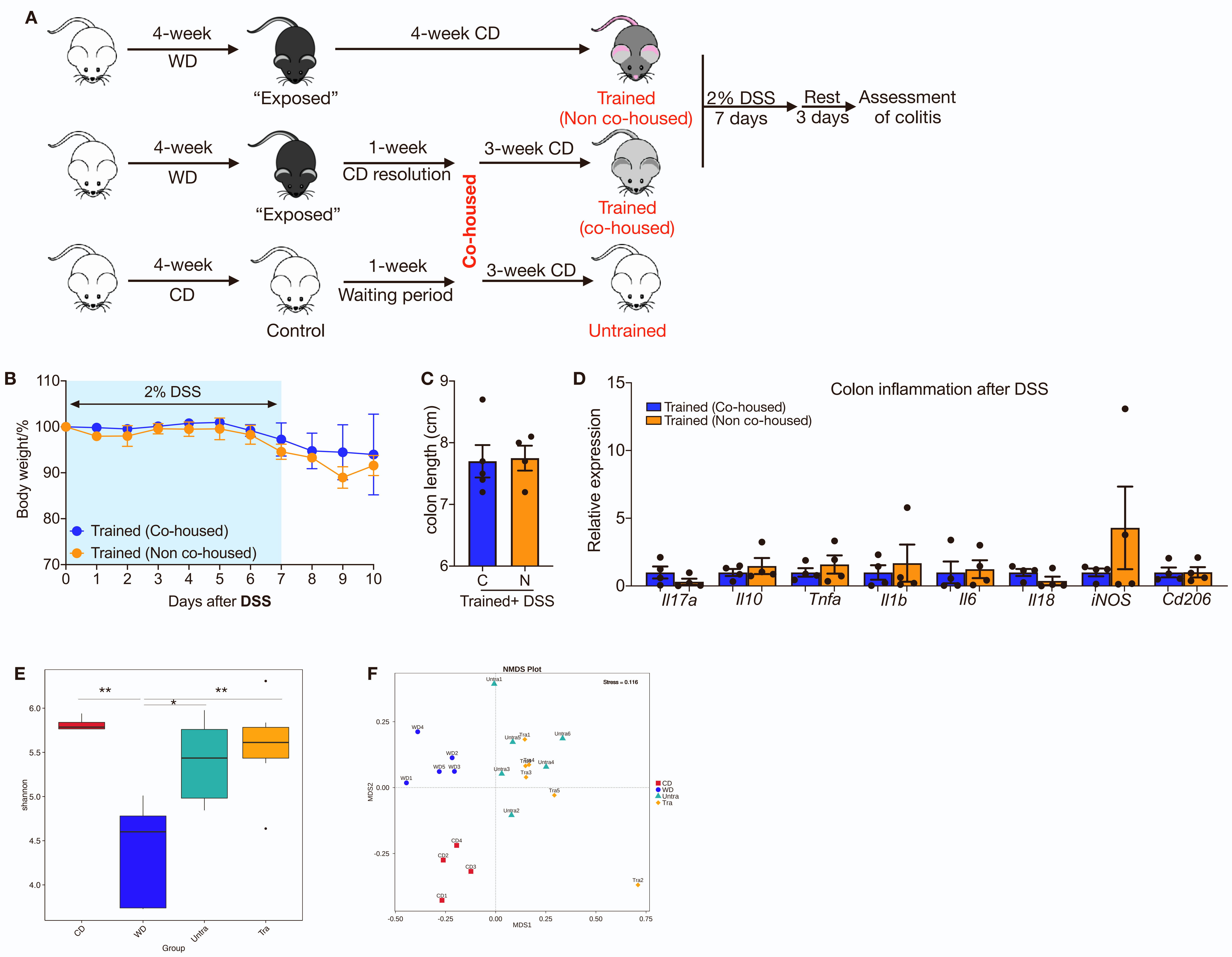
A
4-week
WD
"Exposed"
4-week CD
Trained
(Non co-housed)
2% DSS
7 days
Rest
3 days
Assessment
of colitis
4-week
WD
"Exposed"
1-week
CD resolution
Co-housed
3-week CD
Trained
(co-housed)
4-week
CD
Control
1-week
Waiting period
3-week CD
Untrained
B
2% DSS
Body weight/%
Trained (Co-housed)
Trained (Non co-housed)
Days after DSS
C
colon length (cm)
C
N
Trained+ DSS
D
Colon inflammation after DSS
Trained (Co-housed)
Trained (Non co-housed)
Relative expression
Il17a
Il10
Tnfa
Il1b
Il6
Il18
iNOS
Cd206
E
shannon
Group
CD
WD
Untra
Tra
F
NMDS Plot
Stress = 0.116
MDS1
MDS2
CD
WD
Untra
Tra

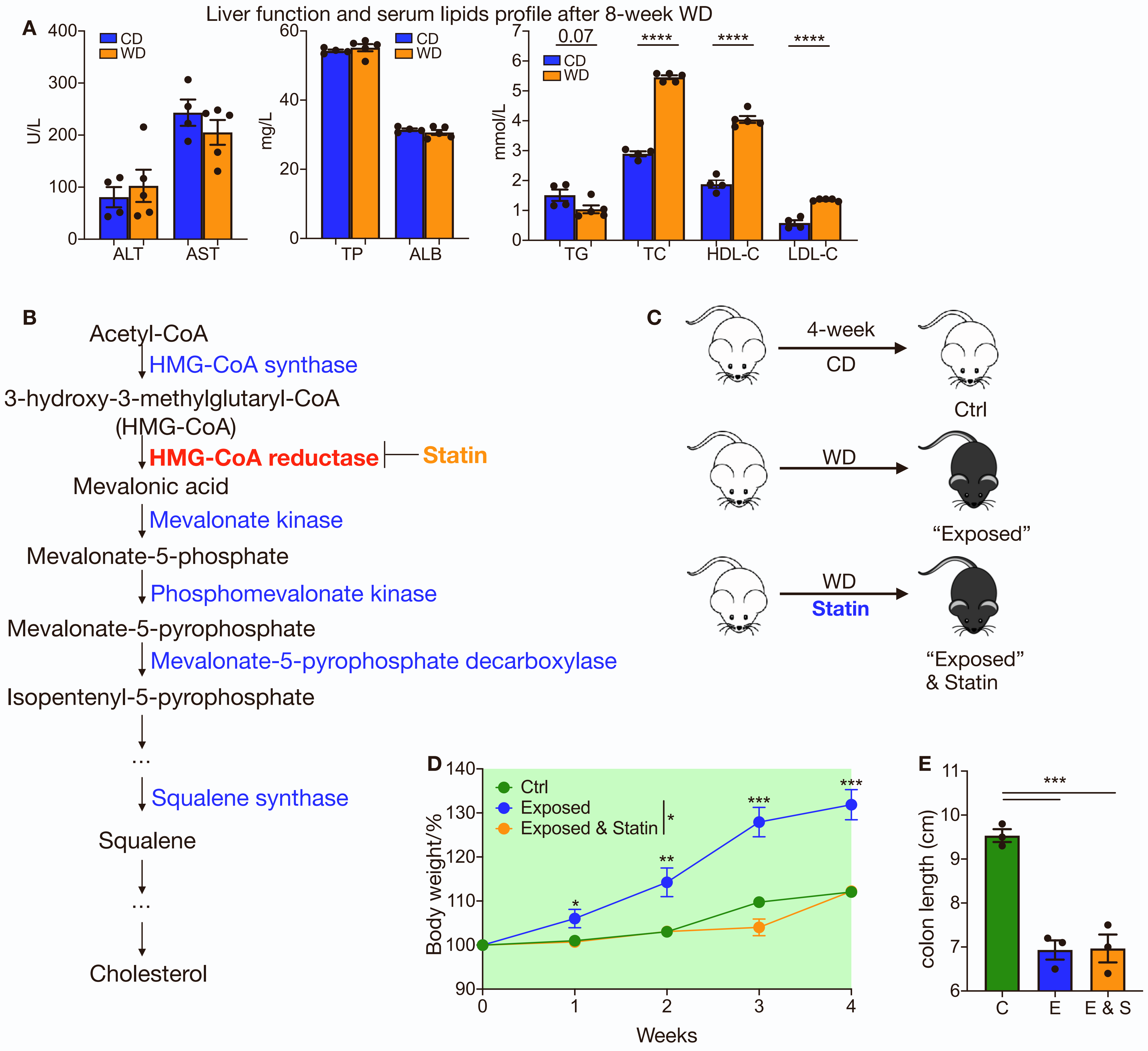

A
Liver function and serum lipids profile after 8-week WD
CD
WD
U/L
ALT
AST
mg/L
TP
ALB
mmol/L
0.07
****
TG
TC
HDL-C
LDL-C
B
Acetyl-CoA
HMG-CoA synthase
3-hydroxy-3-methylglutaryl-CoA
(HMG-CoA)
HMG-CoA reductase
Statin
Mevalonic acid
Mevalonate kinase
Mevalonate-5-phosphate
Phosphomevalonate kinase
Mevalonate-5-pyrophosphate
Mevalonate-5-pyrophosphate decarboxylase
Isopentenyl-5-pyrophosphate
Squalene synthase
Squalene
Cholesterol
C
4-week
CD
Ctrl
WD
"Exposed"
WD
Statin
"Exposed"
& Statin
D
Ctrl
Exposed
Exposed & Statin
Body weight/%
Weeks
E
colon length (cm)
C
E
E & S

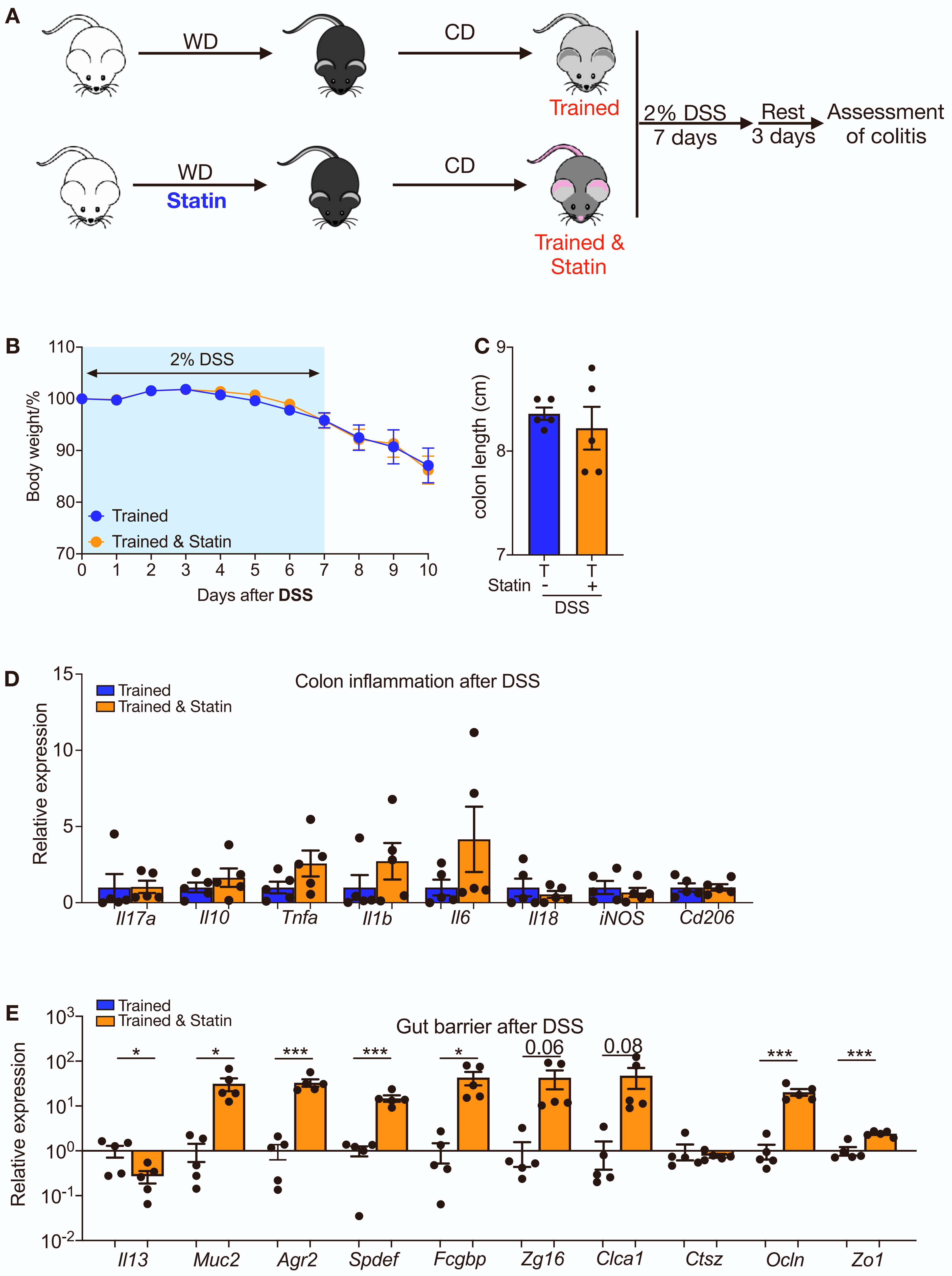
A
WD
CD
Trained
WD
Statin
CD
Trained &
Statin
2% DSS
7 days
Rest
3 days
Assessment
of colitis
B
2% DSS
Body weight/%
Trained
Trained & Statin
Days after DSS
C
colon length (cm)
Statin
DSS
D
Colon inflammation after DSS
Relative expression
Trained
Trained & Statin
Il17a
Il10
Tnfa
Il1b
Il6
Il18
iNOS
Cd206
E
Gut barrier after DSS
Relative expression
Trained
Trained & Statin
0.06
0.08
Il13
Muc2
Agr2
Spdef
Fcgbp
Zg16
Clca1
Ctsz
Ocln
Zo1

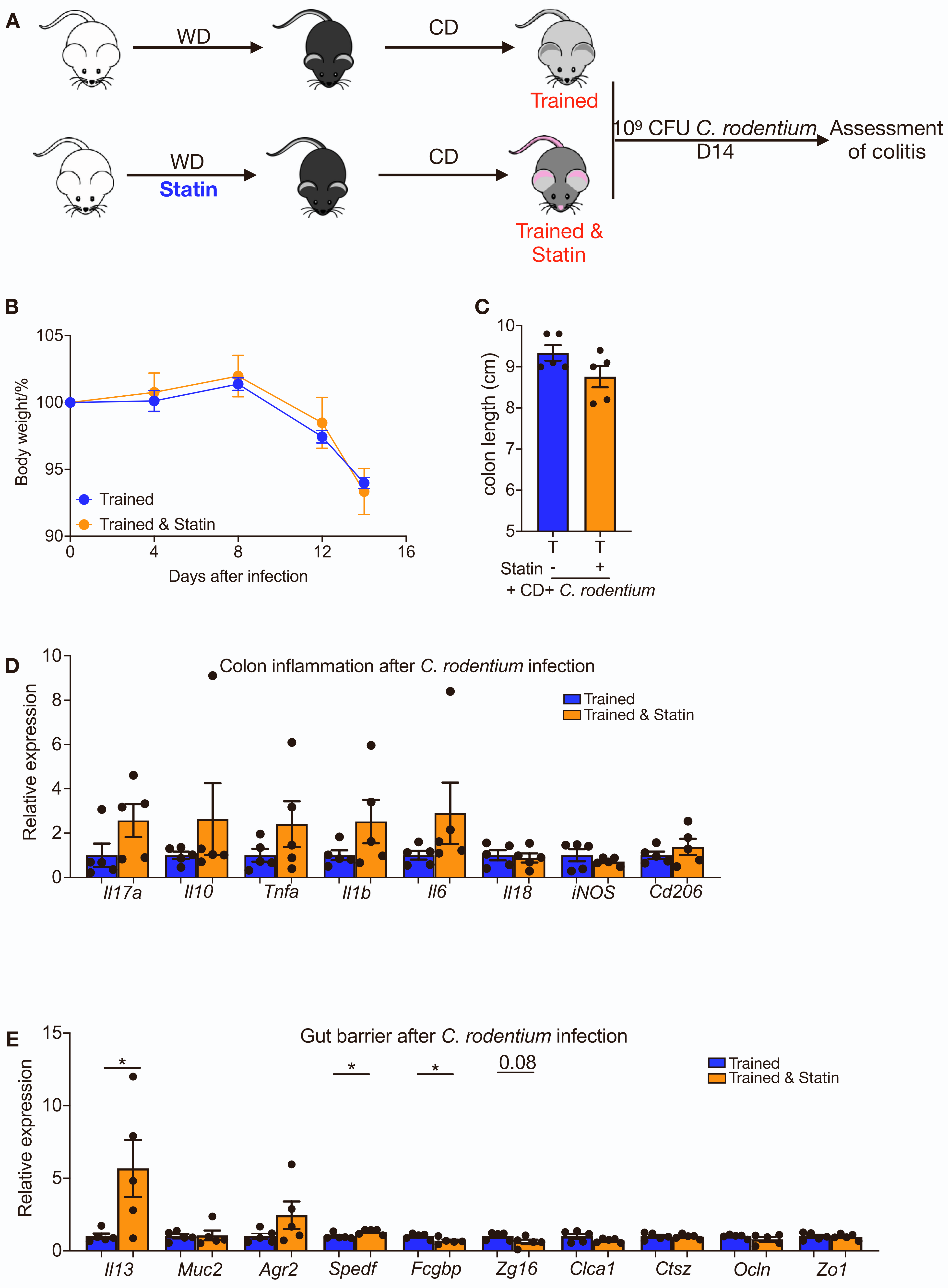

A
WD
CD
Trained
WD
Statin
CD
Trained &
Statin
10⁹ CFU C. rodentium
D14
Assessment
of colitis
B
Body weight/%
Days after infection
Trained
Trained & Statin
C
colon length (cm)
T
T
Statin
-
+
+ CD+ C. rodentium
D
Colon inflammation after C. rodentium infection
Relative expression
Trained
Trained & Statin
Il17a
Il10
Tnfa
Il1b
Il6
Il18
iNOS
Cd206
E
Gut barrier after C. rodentium infection
Relative expression
Trained
Trained & Statin
0.08
Il13
Muc2
Agr2
Spedf
Fcgbp
Zg16
Clca1
Ctsz
Ocln
Zo1

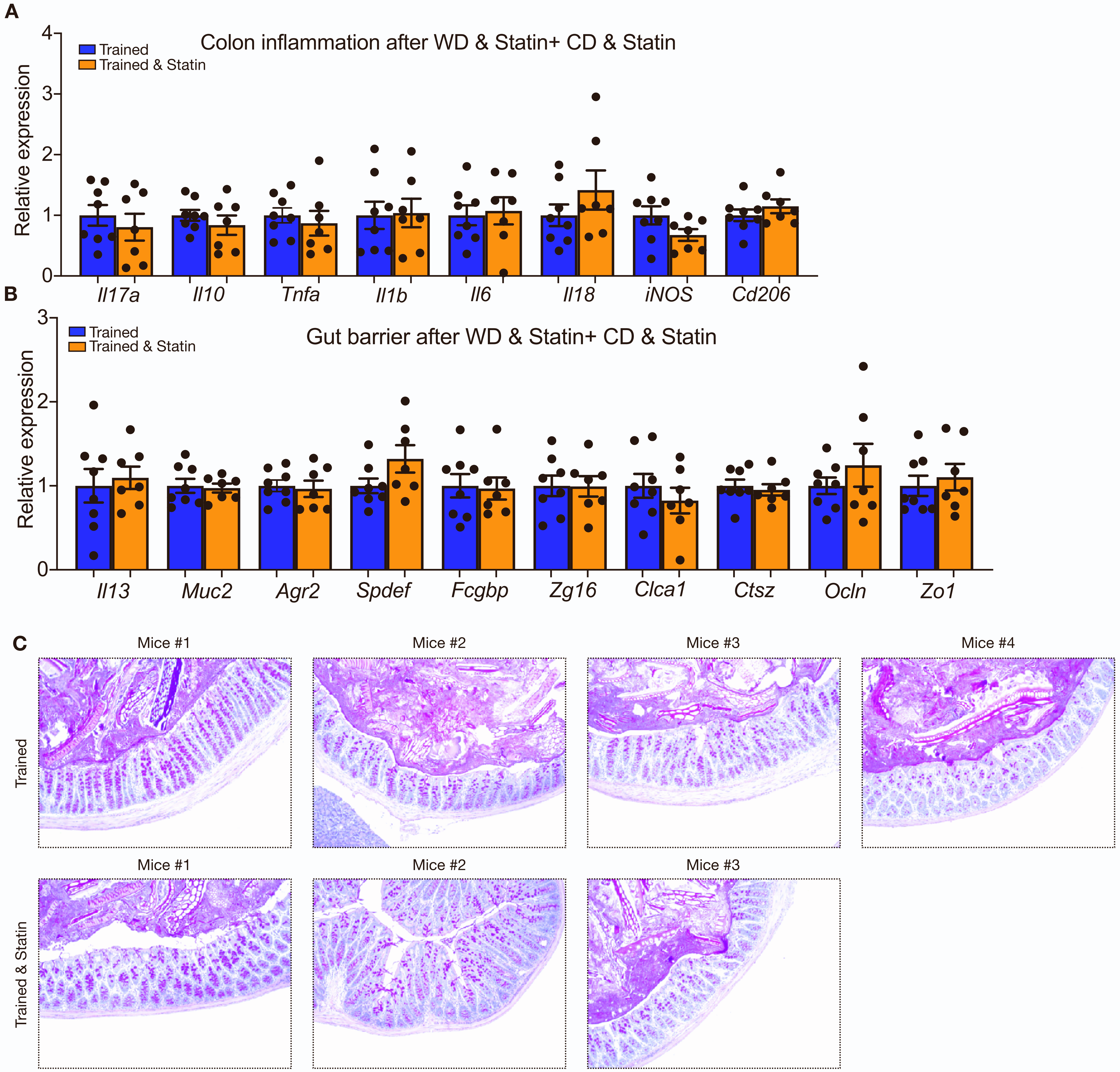
A
Colon inflammation after WD & Statin+ CD & Statin
Trained
Trained & Statin
Relative expression
Il17a
Il10
Tnfa
Il1b
Il6
Il18
iNOS
Cd206
B
Gut barrier after WD & Statin+ CD & Statin
Trained
Trained & Statin
Relative expression
Il13
Muc2
Agr2
Spdef
Fcgbp
Zg16
Clca1
Ctsz
Ocln
Zo1
C
Mice #1
Mice #2
Mice #3
Mice #4
Trained
Trained & Statin

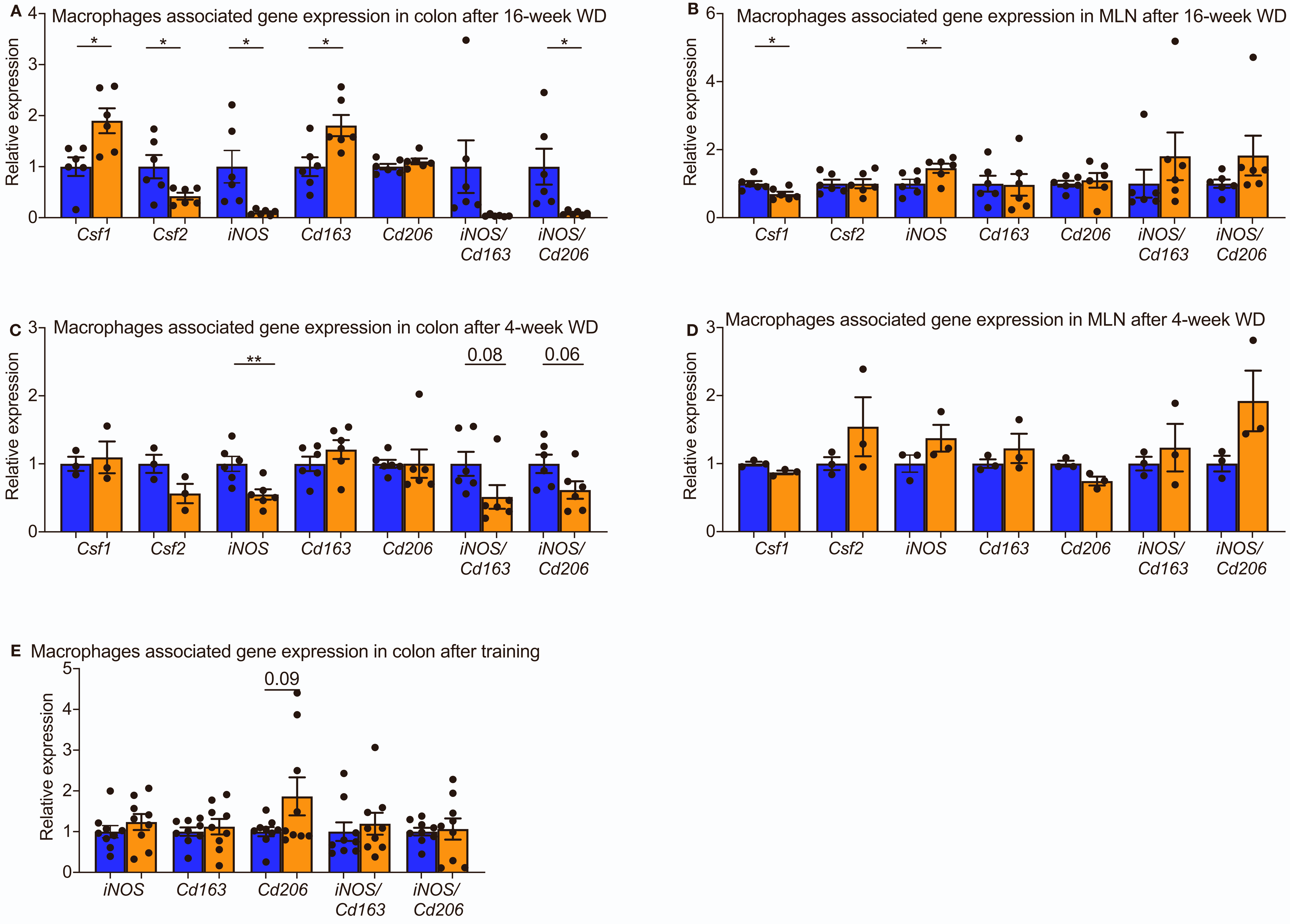

A
Macrophages associated gene expression in colon after 16-week WD
Relative expression
0
1
2
3
4
*
*
*
*
*
Csf1
Csf2
iNOS
Cd163
Cd206
iNOS/Cd163
iNOS/Cd206
B
Macrophages associated gene expression in MLN after 16-week WD
Relative expression
0
2
4
6
*
*
Csf1
Csf2
iNOS
Cd163
Cd206
iNOS/Cd163
iNOS/Cd206
C
Macrophages associated gene expression in colon after 4-week WD
Relative expression
0
1
2
3
**
0.08
0.06
Csf1
Csf2
iNOS
Cd163
Cd206
iNOS/Cd163
iNOS/Cd206
D
Macrophages associated gene expression in MLN after 4-week WD
Relative expression
0
1
2
3
Csf1
Csf2
iNOS
Cd163
Cd206
iNOS/Cd163
iNOS/Cd206
E
Macrophages associated gene expression in colon after training
Relative expression
0
1
2
3
4
5
0.09
iNOS
Cd163
Cd206
iNOS/Cd163
iNOS/Cd206
